# Computation-Guided Placement of Nonfullerene Acceptor Core Halogenation for High-Performance Organic Solar Cells

**DOI:** 10.1021/jacs.5c16058

**Published:** 2026-01-03

**Authors:** Yao Chen, Hongliang Lei, Seunglok Lee, Peihao Huang, Gengsui Tian, Lei Liu, Tianyu Zeng, Changduk Yang, Tainan Duan, Huanyu Zhou, Zeyun Xiao, Tobin J. Marks, Antonio Facchetti

**Affiliations:** † 376348 Chongqing Institute of Green and Intelligent Technology, Chinese Academy of Sciences, Chongqing 400714, P. R. China; ‡ Chongqing School, University of Chinese Academy of Sciences, Chongqing 400714, P. R. China; § School of Energy and Chemical Engineering, 131639 Ulsan National Institute of Science and Technology (UNIST), 50 UNIST-gil, Ulju-gun, Ulsan 44919, South Korea; ∥ Department of Chemistry, the Materials Research Center, Trienens Institute for Sustainability and Energy, 3270 Northwestern University, Evanston, Illinois 60208, United States; ⊥ School of Materials Science and Engineering, 1372 Georgia Institute of Technology, Atlanta, Georgia 30332, United States

## Abstract

The strategic molecular design of nonfullerene acceptors (NFAs) plays a crucial role in enhancing the efficiency of organic solar cells (OSCs). Here, working from first-principles theoretical computation, we report a new series of quinoxaline-based NFAs (**Qx-PhHal**, where Hal = F, Cl, or Br) with varying halogen substitution on the central acceptor core of the molecules to investigate their impact on OSC performance. Notably, OSCs incorporating the brominated NFA demonstrate a significantly higher power conversion efficiency (PCE = 17.58%) than those with fluorinated or chlorinated NFAs (∼14%). Theoretical and experimental analyses reveal that bromination enhances electrostatic interactions, donor–acceptor miscibility, crystallinity, and fibrillar film morphology versus the other halogenated NFAs, thereby enhancing exciton dissociation efficiency, more balanced hole/electron mobility, and reduced exciton recombination rates in the corresponding OSCs. Additionally, ternary solar cells incorporating the brominated NFA as the third component achieve a very high PCE of 20.14%. These findings provide valuable insights into the molecular design of future high-performance NFAs for OSC applications.

## Graphical abstract

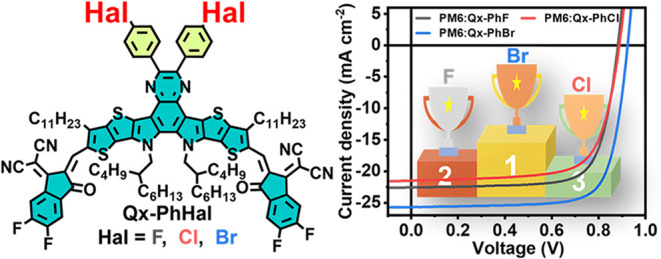

## Introduction

Organic solar cells (OSCs) have garnered considerable research interest for next-generation renewable energy technologies owing to their straightforward scalable solution processability, lightweight, mechanical flexibility, and the absence of toxic heavy metals.^1–5^ Enhancing OSC efficiency has relied on the development of new photoactive materials, innovations in thin-film processing techniques, optimization of device architectures, as well as via fine-tuning of the photoactive blend morphology.^6–12^ Through these innovations, the past decade has witnessed striking progress in OSC power conversion efficiency (PCE), now exceeding 20% mainly through the discovery and optimization of third-generation nonfullerene acceptors (NFAs), especially the development of ITIC, IT-4F, and Y6.^13–26^ Among them, NFAs of the Yn series (Figure 1a) exhibit strong light absorption in the visible–near-infrared (vis–NIR) region, tunable energy levels, excellent charge transport properties, and facile structural modifications, which are key factors for increasing the OSC efficiency.^27–30^ Nevertheless, despite extensive efforts to rationally design NFAs, new approaches to control intermolecular interactions and film morphology, thereby effectively enhancing the PCE by balancing the trade-offs between open-circuit voltage *(V*_OC_), short-circuit current density (*J*_SC_), and fill factor (FF) remains of major interest, yet incompletely explored.^31,32^

**Figure 1:**
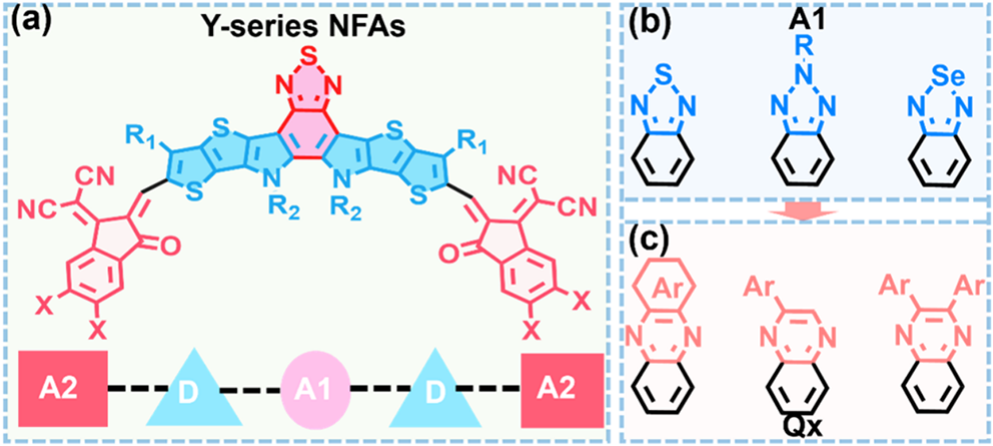
(a) Chemical structure of Y-series NFAs and (b, c) the development of A1 moiety structures reported in the literature.

The Y-series NFAs typically feature an electron-deficient central core, electron-rich donor units, electron-deficient terminal units, and side chains arranged in an acceptor–donor–acceptor–donor–acceptor (A2–D–A1–D–A2) configuration (Figure 1a).^33,34^ Various strategies have been employed to improve the optoelectronic properties of NFAs, including modification of the central subunits, optimization of the acceptor end groups, and engineering the side chains.^35–38^ Among these design approaches, NFA halogenation (e.g., fluorination and chlorination) of various NFA constituents has been particularly effective in enhancing the OSC performance via a combination of electronic and structural packing effects. However, previous halogenation efforts of Y-series NFAs have predominantly focused on the end groups due to the limited functionalization sites on the central (A1) benzothiadiazole core unit (Figure 1b).^39,40^ Importantly, end-group halogenation of benzothiadiazole-centered NFAs, such as those of the Yn family, has shown that F/Cl substitution invariably outperforms its brominated counterparts. For instance, the PCE with various donors of Y6 (X = F) and BTP-eC11 (X = Cl) ranges 15–19% while those of TPIC-4Br (X = Br) are 7–14%, suggesting that Br functionalization does not result in high-performing NFAs.^33,41–44^ Therefore, the state-of-the-art NFAs have widely adopted fluorinated and chlorinated end groups limiting exploration of Br-substituted NFAs.^41,45,46^ Halogenation at the central unit of NFA is very limited with one study suggesting that chlorination outperforms all other types of halogenation^46^ while another suggesting that it is equivalent to bromination;^47^ however, the latter study did not include fluorination. Thus, it remains unclear whether the effect of end-group versus core halogenation differs and whether bromination always underperforms the other halogenation strategies.

Recently, quinoxaline-based (Qx) fused cores have been incorporated into NFA central units (A1) to further improve OSC PCEs (Figure 1c). These cores offer additional versatile functionalization handles, enabling greater flexibility in optimizing energy levels, enhancing intermolecular interactions, and fine-tuning of blend morphology.^48–52^ Indeed, the introduction of the halogens into the Qx-based core has been successful in modifying NFA energy levels and fine-tuning the film morphology, ultimately improving OSC PCE.^47,53^ Recently, Xie et al. investigated the effects of Qx core fluorination position (ortho, meta, and para) on molecular packing and photovoltaic performance.^54^ The para-substituted acceptor (**Qx-p-4F**) shows favorable molecular stacking and, ultimately, higher PCE, providing an alternative avenue for constructing high-performance NFAs. Nevertheless, the role of halogens and the systematic comparison in modulating electrostatic interactions, morphology, and photovoltaic response of NFAs remains unresolved.^47,51^

Encouraged by the success of **Qx-p-4F**, in this contribution, we systematically design, synthesize, characterize, and incorporate in OSCs, a new series of NFAs (**Qx-PhHal**) with varied halogen substitution (Hal = F → Cl → Br) on the phenyl groups attached to the central Qx core to address the role of halogen identity in modulating electrostatic interactions, molecular packing, film morphology, optoelectronic properties, and ultimately the photovoltaic response. The Qx platform enables central-core functionalization that is inaccessible in the widely studied Y series. Here, we specifically investigate how halogen substitution in Qx-based NFAs, while maintaining the end-group halogenation constant, affects structure–property–performance relationships, and whether these trends mirror those reported for Y-series and related NFAs, where F and Cl substitution typically outperform Br.^53,55–60^ Our parallel theoretical calculations suggest that the halogen atoms on the central quinoxaline core have a minimal impact on the individual molecular geometries. However, the dipole moment orientations invert, and the electrostatic potential (ESP) of the halogen sites gradually turns positive on traversing from fluorine to chlorine/bromine, which promotes intermolecular interactions. The optical absorption spectra exhibit a slight blue shift when halogenating from F to Br, accompanied by elevated LUMO levels. Interestingly and unexpectedly, the morphology of the **PM6:Qx-PhHal** OSC blends is modulated by varying the halogen atoms, with bromination affording the most favorable nanofibrillar morphology for charge transport and thereby promoting the highest OSC performance. Consequently, due to the simultaneous enhancement in *V*_OC_, *J*_SC_, and FF, the brominated **PM6:Qx-PhBr** blend exhibits a PCE of 17.58%, far higher than that of the fluorinated **PM6:Qx-PhF** or chlorinated **PM6:Qx-PhCl** blends (PCE ≈ 14%). Importantly, compared to traditional end-group halogenation in other NFAs,^33,41,42,46,47^ an inverse performance trend is observed here, demonstrating that Br can afford more efficient cell performance than the corresponding F/Cl functionalized NFAs. Furthermore, by leveraging the aforementioned advantages, ternary OSCs that incorporate the **Qx-PhBr** NFA as the third component in **PM6:BTP-eC9** devices achieve an impressive PCE of 20.14%. This work highlights the critical role of NFA central-core halogenation in regulating electrostatic interactions and OSC film morphology while revealing an inverse trend in NFA halogenation, thereby providing valuable insights into future molecular design of high-performance NFAs.

## Results and Discussion

### DFT Computations

To gain structural information, density functional theory (DFT) calculations at the B3LYP/6–31G­(d,p) level were performed for the **Qx-PhHal** NFAs prior to their synthesis. From the optimized geometries (Figures 2 and S1–S3), the NFA conjugated backbone is planar with minimal variations upon halogen changes, with the dihedral angles between the halogenated phenyl ring and the Qx core being 39.71, 39.80, and 39.77° for **Qx-PhF**, **Qx-PhCl**, and **Qx-PhBr**, respectively. These comparable dihedral angles and identical backbone planarities for these NFAs suggest that halogen atoms in the para-position have negligible effects on the molecular structure.

**Figure 2:**
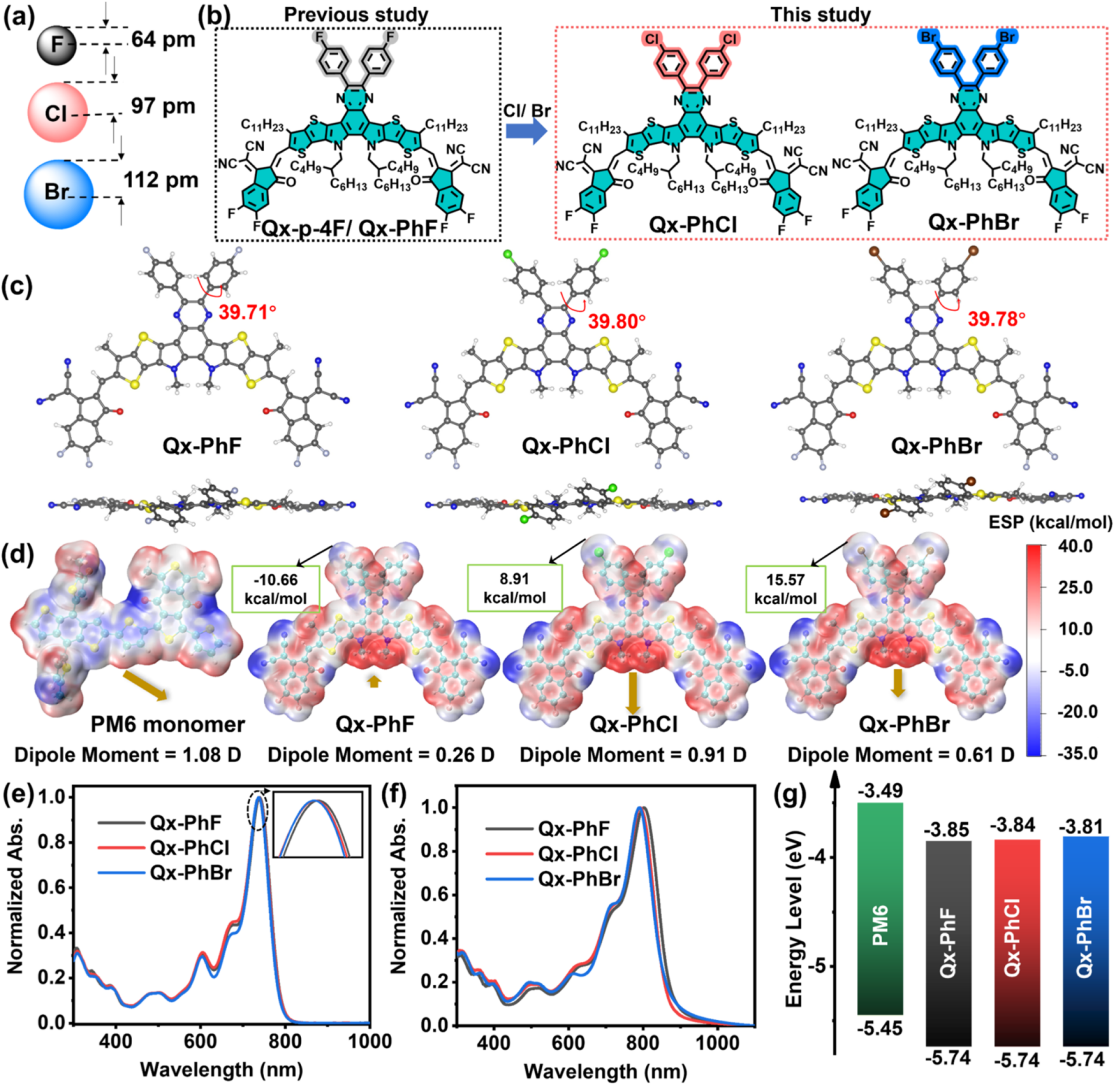
(a) Comparison of halogen atomic radii, (b) chemical structures of **Qx-PhF**, **Qx-PhCl**, and **Qx-PhBr**, (c) DFT-derived molecular structures, (d) electrostatic potential (ESP) and dipole moments of **Qx-PhHal**. (e) UV–vis spectra of the indicated NFAs in dilute solution, (f) in the thin-film state, and (g) energy-level diagrams of **PM6** and the indicated NFAs.

To further evaluate the electronic effects, the ESP distributions and dipole moments of the NFAs were computed and compared. The quantified ESP area distribution and overall average ESP (ESP_avg_) values of the NFA compounds are summarized in Figure S3 and Table S1. Thus, on going from **Qx-PhF**, to **Qx-PhCl**, to **Qx-PhBr**, the ESP area distribution shifts slightly toward the positive direction, leading to an increase in ESP_avg_ from 5.15 to 5.42 to 5.28 kcal mol^–1^, respectively. Considering that most donor polymers have a negative ESP_avg_, e.g., −0.89 kcal mol^–1^ for **PM6**,^61^ replacing F with Cl/Br in **Qx-PhHal** enhances the electrostatic potential difference with the donor polymer, thereby strengthening donor–acceptor intermolecular interactions.^3^ Additionally, the ESP value of the halogen becomes progressively more positive on going from F to Cl and Br, providing more effective interaction sites with the donor polymer. Furthermore, the **Qx-PhHal** dipole moment gradually increases from 0.26 D (**Qx-PhF**) to 0.91 D (**Qx-PhCl**) to 0.61 D (**Qx-PhBr)**, respectively, with reversed dipole moment orientation when replacing F with Cl or Br. The enhanced dipole moment is expected to facilitate more efficient charge dissociation (*vide infra*).

### Synthesis and Characterization

The syntheses of **Qx-PhF**, **Qx-PhCl**, and **Qx-PhBr** are presented in Scheme 1. Detailed characterization of all compounds can be found in the Supporting Information and includes ^1^H NMR and MS analysis. The thermal properties of **Qx-PhHal** NFAs were investigated by differential scanning calorimetry (DSC) and thermogravimetric analysis (TGA) (Figure S4). These new NFAs exhibit high thermal stability with decomposition temperature onsets (*T*_d_) of 323, 318, and 328 °C for **Qx-PhF**, **Qx-PhCl**, and **Qx-PhBr**, respectively, as assessed by TGA. Furthermore, the DSC measurements reveal that the brominated **Qx-PhBr** has a higher melting transition temperature (*T*_m_ = 286 °C) compared to **Qx-PhF** (*T*_m_ = 223 °C) and **Qx-PhCl** (*T*_m_ = 271 °C).

**Scheme 1:**
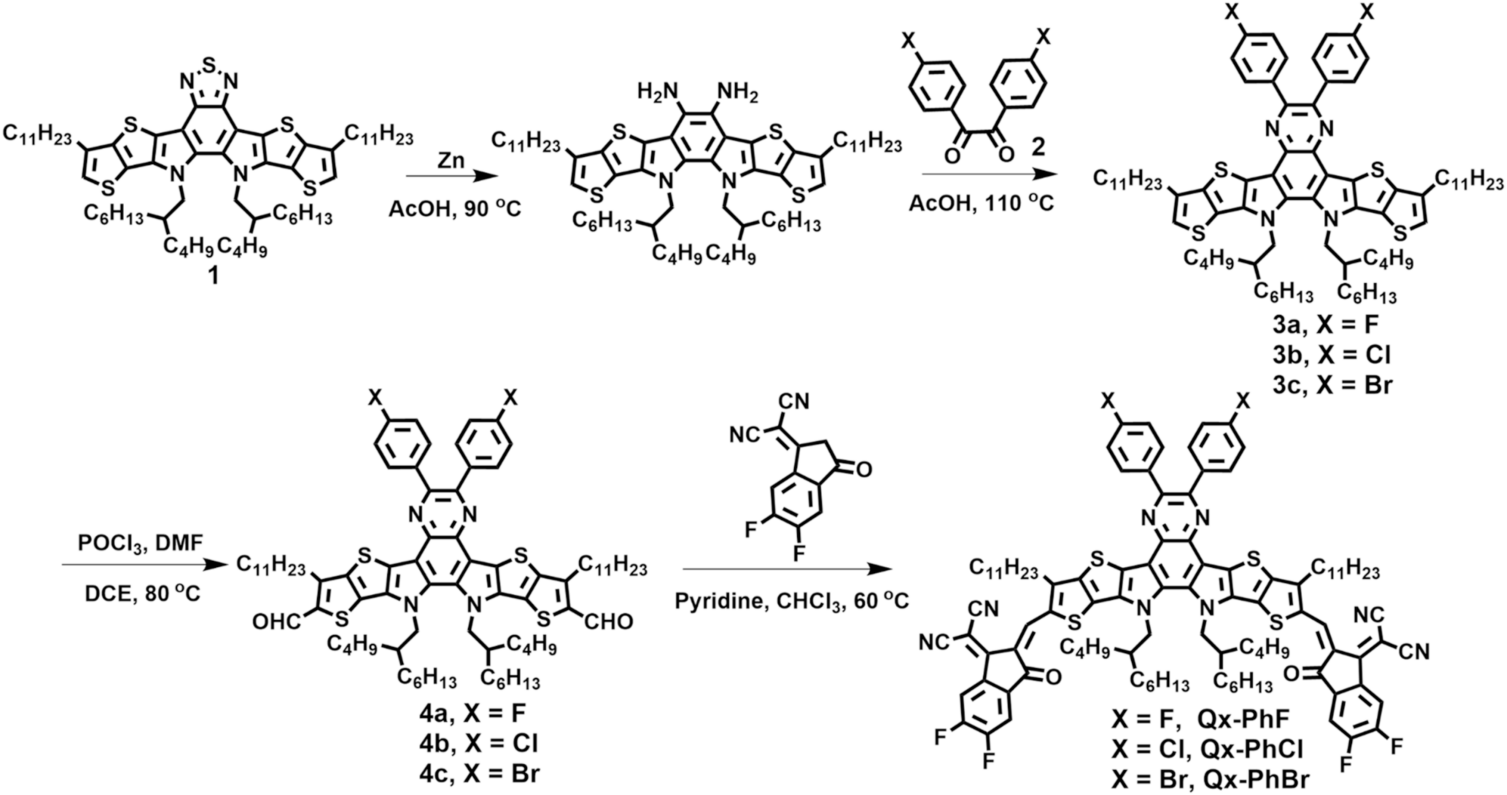
Synthetic Routes to **Qx-PhHal** Acceptors

UV–vis absorption spectra of the present NFAs in dilute chloroform solution and as thin films are shown in Figure 2e,f, and the optical data are summarized in Table 1. The **Qx-PhF**, **Qx-PhCl**, and **Qx-PhBr** optical absorption profiles in solution are almost identical and exhibit only a slightly blue-shifted absorption peak maxima (λ_Max_^Sol.^, 739, 738, and 737 nm, respectively). Going from solution to the solid state, all NFAs exhibit a red-shifted absorption (∼43 nm) with a λ_Max_^Film^ of 802, 792, and 790 nm for **Qx-PhF**, **Qx-PhCl**, and **Qx-PhBr**, respectively, similar to the trend in the solution state. Overall, all NFA exhibit almost identical absorption spectra in solution, while that of the **Qx-PhBr** thin film is slightly blue-shifted compared to that of the **Qx-PhF** one, which may be attributed to the weaker π–π stacking in the solid state (*vide infra*). The optical band gaps (*E*_*g*_^*opt*^) assessed from the onset of thin-film absorption spectra are 1.38, 1.39, and 1.40 eV for **Qx-PhF**, **Qx-PhCl**, and **Qx-PhBr**, respectively. These results reveal that reducing the electronegativity of the halogen atom (F → Br) slightly enlarges the band gap. Thin-film cyclic voltammetry (CV) measurements were next conducted to assess the redox properties and thus the highest occupied molecular orbital (HOMO) and lowest unoccupied molecular orbital (LUMO) energy levels of the newly synthesized NFAs (Figures S5 and 2f). Details are reported in the Supporting Information. Based on the oxidation and reduction onset potentials, the HOMO/LUMO energy levels of **Qx-PhF**, **Qx-PhCl**, and **Qx-PhBr** were determined to be −5.74/–3.85, −5.74/–3.84, and −5.74/–3.81 eV, respectively. Interestingly, while **Qx-PhF**, **Qx-PhCl**, and **Qx-PhBr** have comparable HOMO energies, similar to the trend found from DFT computations, a noticeable upward shift in the LUMO level is observed going from **Qx-PhF** to **Qx-PhCl** to **Qx-PhBr** as the halogen electron-withdrawing ability gradually weakens going from F to Cl to Br, which in principle could raise the *V*_OC_ of the corresponding OSC. Overall, the energy levels of **Qx-PhF**, **Qx-PhCl**, and **Qx-PhBr** are well-aligned with those of the **PM6** donor polymer, which was selected for OSC fabrication.

**Table 1:** Optoelectronic Parameters of **Qx-PhF**, **Qx-PhCl**, and **Qx-PhBr**

NFAs	dipole moment (Debye)	*λ*_max_^sol.^ (nm)	*λ*_max_^Film^ (nm)	*E*_g_^opt^ ^a^ (eV)	*E*_OX_ (V)	*E*_RE_ (V)	E_HOMO_ ^b^ (eV)	E_LUMO_ ^b^ (eV)
**Qx-PhF**	0.26	739	802	1.38	0.94	–0.95	–5.74	–3.85
**Qx-PhCl**	0.91	738	792	1.39	0.94	–0.96	–5.74	–3.84
**Qx-PhBr**	0.61	737	790	1.40	0.94	–0.99	–5.74	–3.81

a Calculated from the empirical formula: *E*_g_ = 1240/λ_onset_.

b Estimated from cyclic voltammetry.

### OSC Fabrication and Performance Evaluation

OSCs were fabricated with the conventional architecture of ITO/PEDOT:PSS/**PM6:Qx-PhF**, **Qx-PhCl**, or **Qx-PhBr**/ PNDIT-F3N/Ag (Figure 3a). The optimal thickness of the photoactive layer is approximately 100 nm. As shown in Tables 2 and S2–S4, the optimized **PM6:Qx-PhBr** blend OSCs deliver a PCE of 17.58% (*V*_OC_ = 0.91 V, *J*_SC_ = 25.59 mA cm^–2^, FF = 75.53%), significantly higher than those of **PM6:Qx-PhF** (PCE = 14.77%, *V*_OC_ = 0.88 V, *J*_SC_ = 22.56 mA cm^–2^, FF = 74.22%) and **PM6:Qx-PhCl** (PCE = 13.59%, *V*_OC_ = 0.89 V, *J*_SC_ = 21.49 mA cm^–2^, FF = 71.21%). Note, all device metrics (*V*_OC_, *J*_SC_, and FF) are simultaneously elevated with the brominated NFA. Thus, in this family an opposite trend in the effect of halogenation is observed compared to traditional end-group halogenated NFAs.^62^ The slightly higher *V*_OC_ of the device utilizing **Qx-PhBr** is attributed to the elevated LUMO energy level. Furthermore, the enlarged *J*_SC_ and FF highly correlate with weaker charge recombination and favorable film morphology (*vide infra*).^63^ As shown in Figure 3c, a similar photoresponse (EQE) is observed for all three blends, which is consistent with the absorption spectra of those blends (Figure 2f). However, the photon-to-electron response intensity of the **PM6:Qx-PhBr** cells is much higher than those of **PM6:Qx-PhF** and **PM6:Qx-PhCl**, with a maximum EQE of 89.7% at 580 nm. The higher integrated current of 24.61 mA cm^–2^ (within 5% error) for **PM6:Qx-PhBr** vs 21.68 mA cm^–2^ for **PM6:Qx-PhF** and 20.80 mA cm^–2^ for **PM6:Qx-PhCl** are aligned with the measured *J*_SC_. Furthermore, the thermal stability of these OSCs was recorded at 80 °C. As shown in Figure S9, the *T*_80_ values (the time needed to reach 80% of the initial PCE) for **PM6:Qx-PhF**, **PM6:Qx-PhCl**, and **PM6:Qx-PhBr** OSCs are 263, 254, and 310 h, respectively, demonstrating that bromination also enhances thermal stability in the devices. Additionally, all OSCs exhibit comparable shelf-storage and light stability (Figure S10).

**Figure 3:**
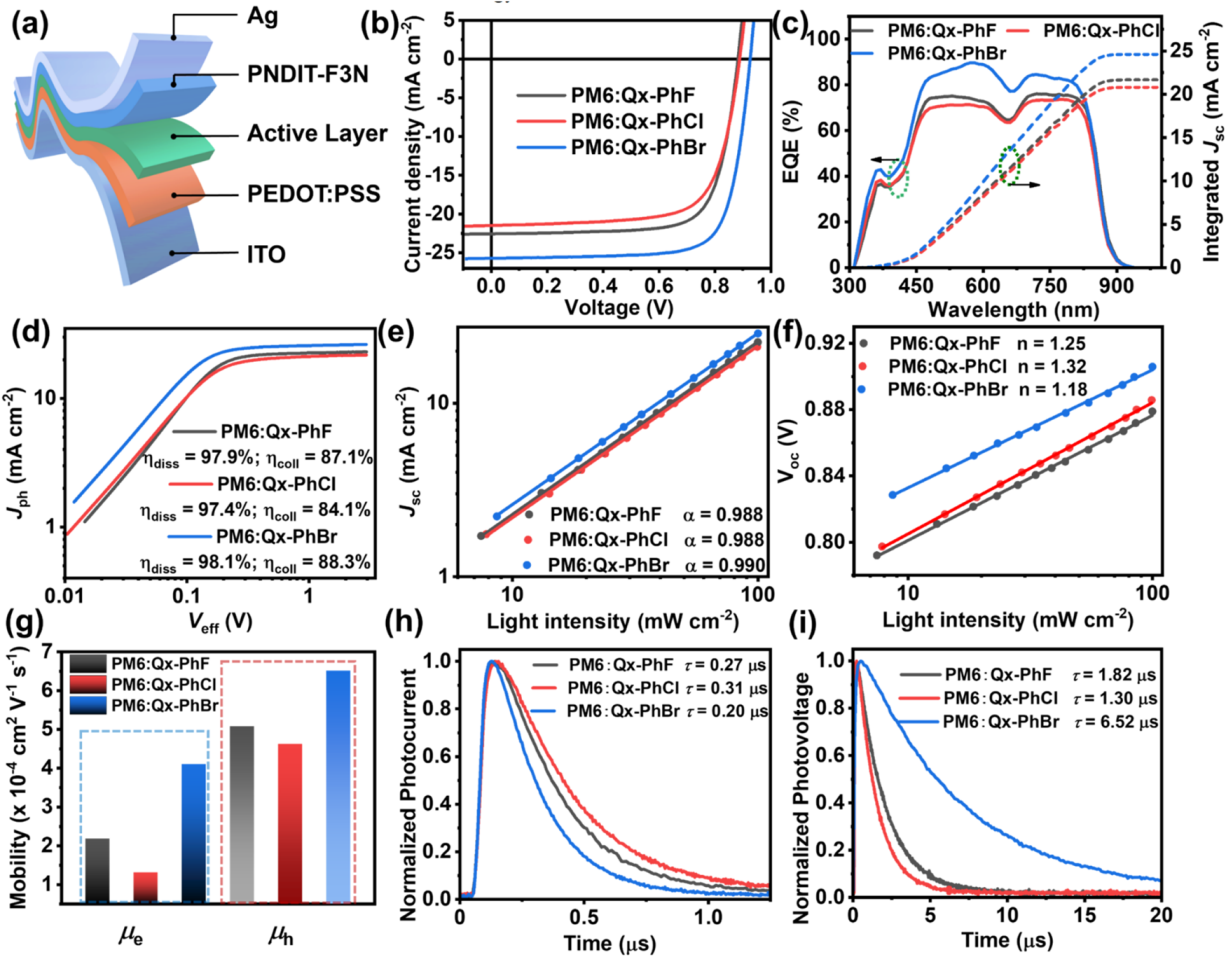
(a) Device architecture of the indicated OSCs characterized in this study; (b) *J*–*V* and (c) EQE curves of the indicated devices; (d) *J*_ph_ versus *V*_eff_ plots; (e) *J*_SC_ versus light intensity; (f) *V*_OC_ versus light intensity; (g) electron and hole mobilities; (h) transient photocurrent; and (i) transient photovoltage of **PM6:Qx-PhF, PM6:Qx-PhCl**, and **PM6:Qx-PhBr** devices.

**Table 2:** Photovoltaic Metrics of **PM6:Qx-PhF**, **PM6:Qx-PhCl**, and **PM6:Qx-PhBr** OSCs

active layer	*V*_OC_ (V)	*J*_SC_ (mA/cm^2^)	*J*_*Cal*_^*EQE*^ ^a^ (mA/cm^2^)	FF (%)	PCE (%)^b^
**PM6:Qx-PhF**	0.88 (0.88 ± 0.002)	22.56 (22.46 ± 0.23)	21.68	74.22 (73.10 ± 0.79)	14.77 (14.45 ± 0.20)
**PM6:Qx-PhCl**	0.89 (0.89 ± 0.002)	21.49 (21.19 ± 0.29)	20.80	71.21 (71.48 ± 0.78)	13.59 (13.42 ± 0.15)
**PM6:Qx-PhBr**	0.91 (0.91 ± 0.004)	25.59 (25.32 ± 0.49)	24.61	75.53 (75.13 ± 0.74)	17.58 (17.20 ± 0.25)

a *J*_*Cal*_^*EQE*^ obtained from the integration of the EQE.

b Average parameters calculated from 10 devices.

### Comparative Analysis of Charge Recombination, Exciton Dissociation, and Charge Transport Properties

Charge generation efficiency was studied by measuring the photocurrent density (*J*_ph_) versus the effective voltage (*V*_eff_) (Figure 3d).^64,65^ Thus, the efficiency of exciton dissociation (η_diss_) of the **PM6:Qx-PhBr** solar cells is slightly larger than that of the other two blends, with η_diss_ values of 97.9%, 97.4%, and 98.1% for **PM6:Qx-PhF**, **PM6:Qx-PhCl**, and **PM6:Qx-PhBr**, respectively. Similarly, the efficiency of charge collection (η_coll_) of the **PM6:Qx-PhBr** OSCs is greater than that of the other two blends with η_coll_ values of 87.1, 84.1, and 88.3% for **PM6:Qx-PhF**, **PM6:Qx-PhCl**, and **PM6:Qx-PhBr**, respectively. The greater η_diss_ and η_coll_ in the **PM6:Qx-PhBr** blend indicates that the exciton dissociation and charge collection are greatly enhanced by central-core bromination, thereby elevating the PCE of the OSC.^66^

Furthermore, light intensity (*I*) dependent *J*–*V* analysis of the devices was next carried out to investigate bimolecular recombination. Typically, the relationship between *J*_SC_ and *I* is expressed as *J*_SC_ ∝ *I* ^α^, where the α value represents the efficiency of bimolecular recombination.^67,68^ A value of 1 for α suggests the absence of bimolecular recombination loss.^28,69^ Interestingly, all blends exhibit a comparable α of 0.99, indicating the absence of bimolecular recombination in these halogenated NFA blends (Figure 3e). The reduced bimolecular recombination in the **PM6:Qx-PhBr** blend correlates with its higher FF of 75.53% than others. To obtain deeper insight into other OSC recombination processes, the *I*-depended *V*_OC_ was evaluated, which is governed by the relation *V*_OC_ = *n ×* (*k*_B_ *T*/*e*) *×* ln­(*I*)+*C*, where *n* is the ideality constant, *k*_B_ is the Boltzmann constant, *T* is the temperature, *e* is the elementary charge, and *C* is a constant.^69,70^ A smaller slope (close to 1.0 *k*_B_ *T*/e) indicates reduced monomolecular recombination in OSCs, typically associated with suppressed trap-assisted recombination.^71^ The extracted *n* values were found to be 1.25, 1.32, and 1.18 for **PM6:Qx-PhF**, **PM6:Qx-PhCl**, and **PM6:Qx-PhBr**, respectively, indicating that the **PM6:Qx-PhBr** blend effectively suppresses the trap-assisted recombination (Figure 3f). This finding demonstrates that central-core halogenation engineering of **Qx-PhHal** NFAs strongly affects bimolecular recombination and trap-assisted charge recombination with the brominated central core more efficiently suppressing charge recombination.

Charge transport is another critical property connected with PCE variations.^4^ Therefore, the blend hole and electron^72–74^ mobilities of the blend were measured using the space–charge-limited current (SCLC) method^75^ (Figures 3g and S11) with all values summarized in Table S4. The **PM6:Qx-PhBr** blend film exhibits a higher hole mobility (μ_h_) of 6.51 × 10^–4^ cm^2^ V^–1^ s^–1^ and electron mobility (μ_e_) of 4.10 × 10^–4^ cm^2^ V^–1^ s^–1^ than those of both **PM6:Qx-PhF** and **PM6:Qx-PhCl** (μ_h_ = 5.08 × 10^–4^ and 4.62 × 10^–4^ cm^2^ V^–1^ s^–1^; μ_e_ = 2.18 × 10^–4^ and 1.31 × 10^–4^ cm^2^ V^–1^ s^–1^, respectively). Importantly, **PM6:Qx-PhBr** has a more balanced charge carrier mobility ratio (μ_e_/μ_h_ = 0.63) than those of **PM6:Qx-PhF** and **PM6:Qx-PhCl** (μ_e_/μ_h_ = 0.43 and 0.28, respectively) blends. The enhanced and more balanced electron/hole mobility in the **PM6:Qx-PhBr** blend contributes to more favorable charge extraction and charge transport, and thus, higher FF and PCE are obtained. Next, to gain a deeper understanding of the charge recombination and extraction behaviors, transient photocurrent (TPC) and transient photovoltage (TPV) evaluations were conducted.^76^ The charge extraction time and carrier lifetime were extracted from the TPC and TPV decay dynamics through monoexponential fits. As shown in Figure 3h, the **PM6:Qx-PhBr** OSCs exhibit a slightly shorter charge extraction time of 0.20 μs than those of the **PM6:Qx-PhF** (0.27 μs) and **PM6:Qx-PhCl** (0.31 μs) blends, indicating that the **PM6:Qx-PhBr**-based OSCs extract charge carriers more rapidly. Furthermore, the carrier lifetimes (τ) derived from the TPV measurements (Figure 3i) are significantly longer for the **PM6:Qx-PhBr** blend at 6.52 μs versus 1.82 and 1.30 μs for **PM6:Qx-PhF** and **PM6:Qx-PhCl**-based devices, respectively, consistent with reduced charge recombination in the **PM6:Qx-PhBr** devices. Consequently, more efficient charge extraction and reduced free charge recombination explain the higher *J*_SC_ and FF values (see details in Table 2) observed in the **PM6:Qx-PhBr** OSCs.

### Thin-Film Morphology and Microstructure Analysis

To gain a comprehensive understanding of the morphology, atomic force microscopy (AFM) and transmission electron microscopy (TEM) were applied to elucidate phase separation in the active layer. AFM imaging (Figures 4 and S12) reveals that the **PM6:Qx-PhF**, **PM6:Qx-PhCl**, and **PM6:Qx-PhBr** blends exhibit a uniform surface morphology with root-mean-square (RMS) roughnesses of 1.81, 1.39, and 1.79 nm, respectively. However, compared with the **PM6:Qx-PhF** film, the **PM6:Qx-PhCl** and **PM6:Qx-PhBr** blends possess relatively pronounced nanofibril morphologies. Interestingly, the brominated **PM6:Qx-PhBr** thin film exhibits a larger diameter fiber structure in comparison with that of **PM6:Qx-PhCl** (∼27 vs. ∼22 nm), resulting in a favorable phase-separated morphology (Figures S12–S13).^77,78^ These results indicate that the **PM6:Qx-PhBr** blend has an ideal morphology that is beneficial for exciton dissociation and charge transport, in conformity with the aforementioned reduced recombination loss and enhanced charge mobility.

**Figure 4:**
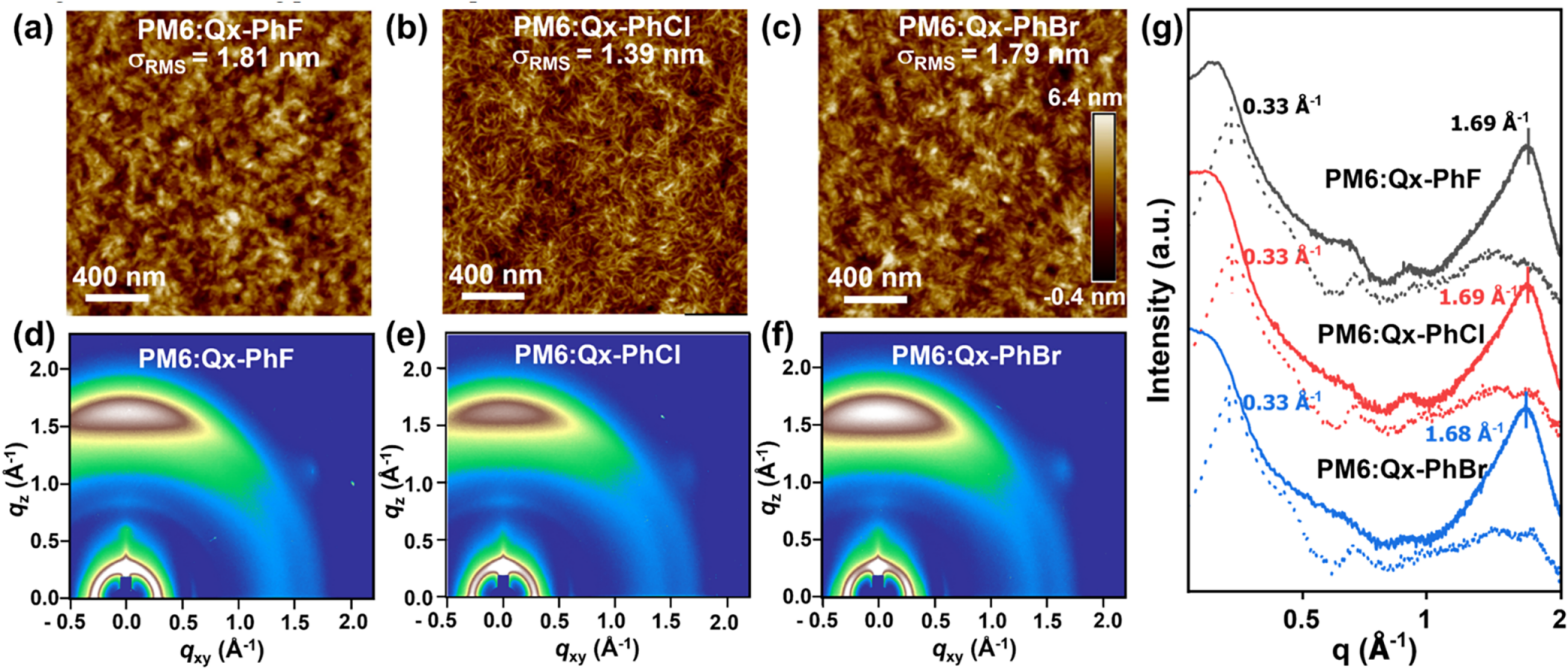
(a–c). AFM height images, (d–f) 2D GIWAXS patterns of the indicated blend films, and (g) corresponding line-cut profiles of the indicated blend films.

Grazing-incidence wide-angle X-ray scattering (GIWAXS) was next employed to investigate the molecular packing and crystallinity of the neat NFAs and their corresponding blend films. The GIWAXS patterns and the corresponding line cuts for the blend films are provided in Figures 4d–g and S14–S15, and all data are summarized in Tables S6–S9. As shown in Figure S14, the neat NFAs exhibit a preferential π-face-on orientation of the conjugated backbone with respect to the substrate surface, as evidenced by the strong π–π (010) stacking peak at around *q*_*x*_ = 1.64–1.69 Å^–1^ in the out-of-plane (OOP) direction and a diffraction peak of (100) at around *q*_*xy*_ = 0.37–0.38 Å^–1^ in the in-plane (IP) direction. From the in-plane (100) reflection (*q*_*xy*_), it is found that when the halogen atomic number increases from F to Br, the lamellar *d*-spacing (d_l_) contracts from 17.1 Å (**Qx-PhF**), to 16.4 Å (**Qx-PhCl**) and 16.4 Å (**Qx-PhBr**), and the crystal coherence length (CCL) falls from 72.4 to 33.6 to 42.0 Å. For the (010) out-of-plane reflections, the π–π intermolecular *d*-spacing distance (*d*_π_) is similar within the range from 3.7 to 3.8 Å. The GIWAXS data for the blend films indicate similar preferential face-on π–π intermolecular stacking distributions of the donor polymer chains with respect to the substrate surface (Figure 4), as evidenced by the strong π–π (010) stacking peak at around *q*_*x*_ = 1.68–1.69 Å^–1^ in the OOP direction and a diffraction peak of (100) at around *q*_*xy*_ ≈ 0.33 Å^–1^ in the IP direction. For the **PM6:Qx-PhF**, **PM6:Qx-PhCl**, and **PM6:Qx-PhBr** blends, the reflections located at *q*_*xy*_ ≈ 0.33 Å ^–1^ are attributed to the donor polymer (100) alkyl-chain stacking. Upon increasing the halogen atomic radius in the blend (**F** → **Br**), the d_l_ remains constant at ∼19 Å, while the CCL monotonically increases from 95.3 Å (**PM6:Qx-PhF**), 95.8 Å (**PM6:Qx-PhCl**) to 106.1 Å (**PM6:Qx-PhBr**). For the out-of-plane direction, almost identical π–π stacking (010) diffraction peaks are found at *q*_*z*_ = 1.68–1.69 Å^–1^. The calculated *d*_π_ values are 3.72, 3.72, and 3.75 Å, and the corresponding CCL values are 22.7 Å for **PM6:Qx-PhF**, 22.7 Å for **PM6:Qx-PhCl**, and 21.6 Å for **PM6:Qx-PhBr** blend. However, the π-stacking intensity gradually increases from **PM6:Qx-PhF** to **PM6:Qx-PhBr**, suggesting a greater order.

Additionally, in order to qualitatively assess the D–A miscibility, the water and formamide contact angles of donors and acceptors were measured to calculate the surface tension (γ) and Flory–Huggins interaction parameter (χ_donor–acceptor_) (Figure S16 and Table S8). The aqueous contact angles of **PM6**, **Qx-PhF**, **Qx-PhCl**, and **Qx-PhBr** films are 106.82°, 102.10°, 96.65°, and 96.36°, respectively, while the formamide contact angles are 84.33, 74.78, 74.74, and 74.44°, respectively, resulting in γ for **PM6**, **Qx-PhF**, **Qx-PhCl**, and **Qx-PhBr** films of 22.56, 31.38, 26.48, and 26.64 mN m^–1^, respectively. Therefore, the calculated χ value of the blends with **PM6** are 0.73, 0.16, and 0.16 for **PM6:Qx-PhF**, **PM6:Qx-PhCl**, and **PM6:Qx-PhBr**, respectively, implying a far greater miscibility of **Qx-PhCl** and **Qx-PhBr** with **PM6**. These results demonstrate that the halogenation of the central unit of **Qx-PhHal** NFAs greatly affects the D–A miscibility, affording a heretofore unrecognized opportunity to manipulate OSC morphologies favoring a nanofibril morphology that enhances optoelectronic properties.^79,80^

Finally, the ESP of **PM6** and new synthesized NFAs **Qx-PhF**, **Qx-PhCl**, and **Qx-PhBr** were computed to probe how the intermolecular interaction affects the charge generation and recombination (Figure S3). Although the molecular conformation minimally changes upon different NFA halogenations, the difference in ESP between **Qx-PhBr** and **PM6** monomer is larger than that of **Qx-PhF** or **Qx-PhCl** and **PM6**. It is well-recognized that larger differences in ESP between the donor and acceptor lead to stronger intermolecular interactions,^81^ which aligns with the present superior miscibility of **Qx-PhBr** and **PM6** and the aforementioned photovoltaic findings.

Molecular dynamics simulations of **PM6:Qx-PhF**, **PM6:Qx-PhCl**, and **PM6:Qx-PhBr** blends were carried out (Figure S17). In the **PM6:Qx-PhBr** blend, the first peaks of the D–D g­(r) curves appear at a greatly shorter distance compared to those in the **PM6:Qx-PhF** and **PM6:Qx-PhCl** blends. This data corroborate enhanced molecular packing as well as stronger intermolecular interactions within the donor and acceptor domains in the former blend, respectively, which is the key characteristic for promoting more ordered structures with enhanced charge transport. Additionally, the first A–A and D–A g­(r) peak in **PM6:Qx-PhCl** and **PM6:Qx-PhBr** is located at a shorter distance than that in **PM6:Qx-PhF**, implying stronger acceptor–acceptor and donor–acceptor interactions in the former blends. These results are aligned with the morphology characterizations.

### Ternary Solar Cells

The ternary formulation of mixing acceptors is a common strategy to further improve OSC performance.^53,82^ As a high-efficiency binary OSC system, **PM6:BTP-eC9** has garnered considerable attention. Therefore, we introduced brominated NFA **Qx-PhBr** into **PM6:BTP-eC9** as the third component to demonstrate that further PCE enhancement is possible (Figure S18). The optimal *J–V* curves for the binary and ternary systems are shown in Figure 5, and the device parameters are summarized in Table S11. The control binary device based on **PM6:BTP-eC9** exhibits a PCE of 19.03% with a *V*_OC_ = 0.85 V, *J*_SC_ = 28.78 mA cm^–2^, and FF = 77.38%, which is consistent with the literature values.^83^ Upon incorporation of **Qx-PhBr** into the **PM6:BTP-eC9** blend, the resulting ternary device exhibits an elevated *V*_OC_ of 0.86 V, *J*_SC_ of 29.40 mA cm^–2^, and FF of 79.50%, thus delivering a remarkable PCE of 20.14%. All devices exhibit broad photoresponse ranging from 300 to 900 nm (Figures 5 and S19). Compared to the binary OSCs, the ternary OSCs with **Qx-PhBr** exhibit relatively higher photoresponse, which contributes to the enhanced *J*_*SC*_ values. Moreover, the *J*_SC_ values calculated from the integration of the EQE spectra are 27.57 and 28.14 mA cm^–2^, respectively, in alignment with the corresponding *J–V* curves (within 5% mismatch). Additionally, the thermal stability of these OSCs was recorded at 80 °C, and the corresponding binary and ternary OSCs exhibit identical PCE loss under this thermal stress condition (Figure S17). Additionally, despite the larger PCEs, the incorporation of **Qx-PhBr** into the **PM6:BTP-eC9** blend slightly improves the storage stability and light stability (Figure S21). These results highlight the suitability of a brominated NFA for enhancing the ternary photovoltaic performance.

**Figure 5:**
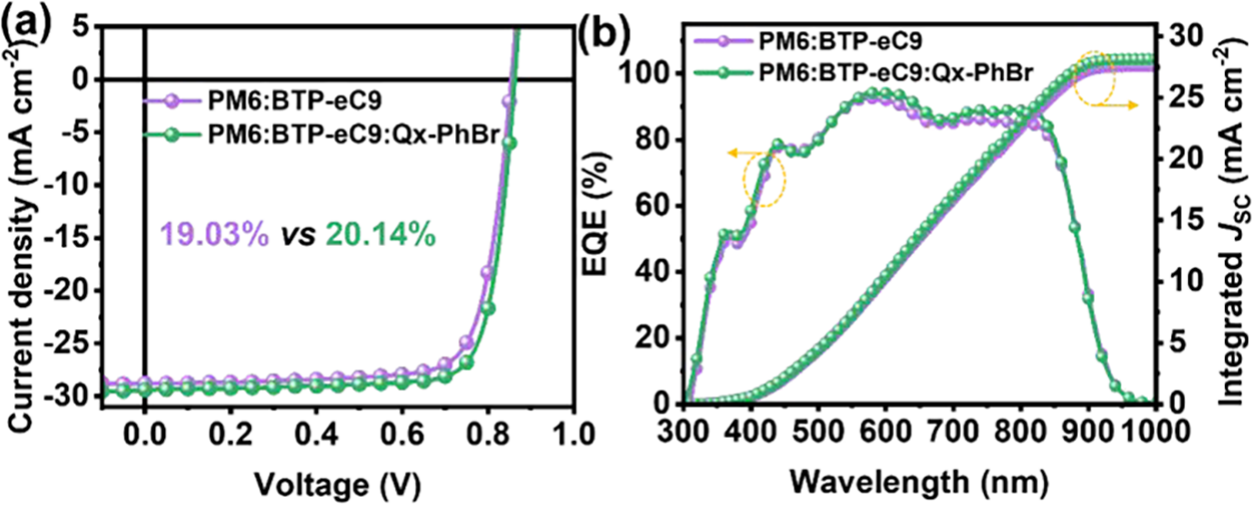
(a) *J*–*V* and (b) EQE curves of the indicated binary and ternary devices.

## Conclusions

A series of **Qx-PhHal** NFAs systematically functionalized with the halogen atoms F, Cl, and Br central cores were characterized to understand how halogen substitution affects the electrostatic interactions, film microstructures, and OSC performance. Interestingly, the bromine-containing **PM6**:**Qx-PhBr** devices exhibit a higher PCE than those of the **PM6**:**Qx-PhF** and **PM6**:**Qx-PhCl** OSCs (17.58 vs 14.77% and 13.59%), which correlates with the enhanced *V*_OC_ (0.91 vs 0.88 and 0.89 V), *J*_SC_ (25.59 vs 22.56 and 21.49 mA cm^–2^), and FF (75.53 vs 74.22% and 71.21%). DFT computations reveal that bromination promotes blend electrostatic interactions, and film morphological analysis indicates that **PM6**:**Qx-PhBr** blends exhibit a pronounced nanofibril morphology. Detailed analysis of exciton dissociation, carrier recombination, D–A miscibility, and charge transport reveals that **PM6**:**Qx-PhBr** exhibits a larger and more balanced μ_e_/μ_h_, more efficient exciton dissociation, enhanced charge collection capacity, and reduced bimolecular recombination compared to the analogous **PM6**:**Qx-PhF** and **PM6**:**Qx-PhCl** blends. Finally, ternary solar cells employing **Qx-PhBr** as the third component realize a PCE of 20.14%. This study began with theoretical computation to systematically investigate the impact of various halogen substitutions at the central core, leading to the development of high-performance OSCs. These results highlight the vital role and versatility of fine-tuning acceptor structures, particularly through the incorporation of heavier halogens.

## Supplementary Material



## Notes

Experimental section, materials synthesis, NMR spectra and mass spectra, cyclic voltammetry, thermogravimetric analysis, DFT calculation, grazing-incidence wide-angle X-ray scattering, solar cell device stability, space–charge-limited current measurement, effective voltage and light intensity measurements, contact angle measurement, miscibility analyses, and references (PDF)
