# Supplementary material for: Computation-Guided Placement of Nonfullerene Acceptor Core Halogenation for High-Performance Organic Solar Cells
Source: J Am Chem Soc. 2026 Jan 3;148(1):723–33. doi: 10.1021/jacs.5c16058 (PMC12814343; doi:10.1021/jacs.5c16058)
Supplement: Supplementary file 1 [file ja5c16058_si_001.pdf]

Supporting Information

Computation-Guided Placement of Nonfullerene Acceptor Core Halogenation for High-Performance Organic Solar Cells

Yao Chen[a, b*], Hongliang Lei[a, b], Seunglok Lee[c], Peihao Huang[a, b], Gengsui Tian[a, b], Lei Liu[a, b], Tianyu Zeng[a, b], Changduk Yang[c], Tainan Duan[a, b], Huanyu Zhou[e], Zeyun Xiao[a, b*], Tobin J. Marks[d*], and Antonio Facchetti[d, e*]

[a] Chongqing Institute of Green and Intelligent Technology, Chinese Academy of Sciences, Chongqing 400714, P. R. China.

[b] Chongqing School, University of Chinese Academy of Sciences, Chongqing 400714, P. R. China

[c] School of Energy and Chemical Engineering, Ulsan National Institute of Science and Technology (UNIST), 50 UNIST-gil, Ulju-gun, Ulsan 44919, South Korea

[d] Department of Chemistry, the Materials Research Center, Trienens Institute for Sustainability and Energy, Northwestern University, Evanston, Illinois 60208, United States.

[e] School of Materials Science and Engineering, Georgia Institute of Technology, Atlanta, Georgia 30332, United States

*Corresponding author

Email addresses: chenyao@cigit.ac.cn, xiao.z@cigit.ac.cn, t-marks@northwestern.edu, antonio.facchetti@mse.gatech.edu

Contents

1. **Density functional theory (DFT) computations** S3

2. **Synthesis** S6

3. **Thermal analyses** S10

4. **Photophysical property characterizations** S10

5. **Electrochemistry property characterizations** S11

6. **Fabrication of organic solar cells** S12

7. **Stability** S16

8. **Mobility measurements** S17

9. **Transient photovoltage (TPV) and transient photocurrent (TPC)** S18

10. **AFM and TEM measurements** S19

11. **GIWAXS measurements** S20

12. **Contact angle measurements** S23

13. **Molecular dynamics simulation** S24

14. **Ternary solar cells** S25

15. **NMR spectra and mass analyses of the indicated materials** S28

16. **References** S34

1. Density functional theory (DFT) computations

The molecular geometries, energy levels, and dipole moments are computed by Gaussian 09 (B3LYP/6-311G (d, p) level), where the alkyl side chains are simplified to methyl groups.

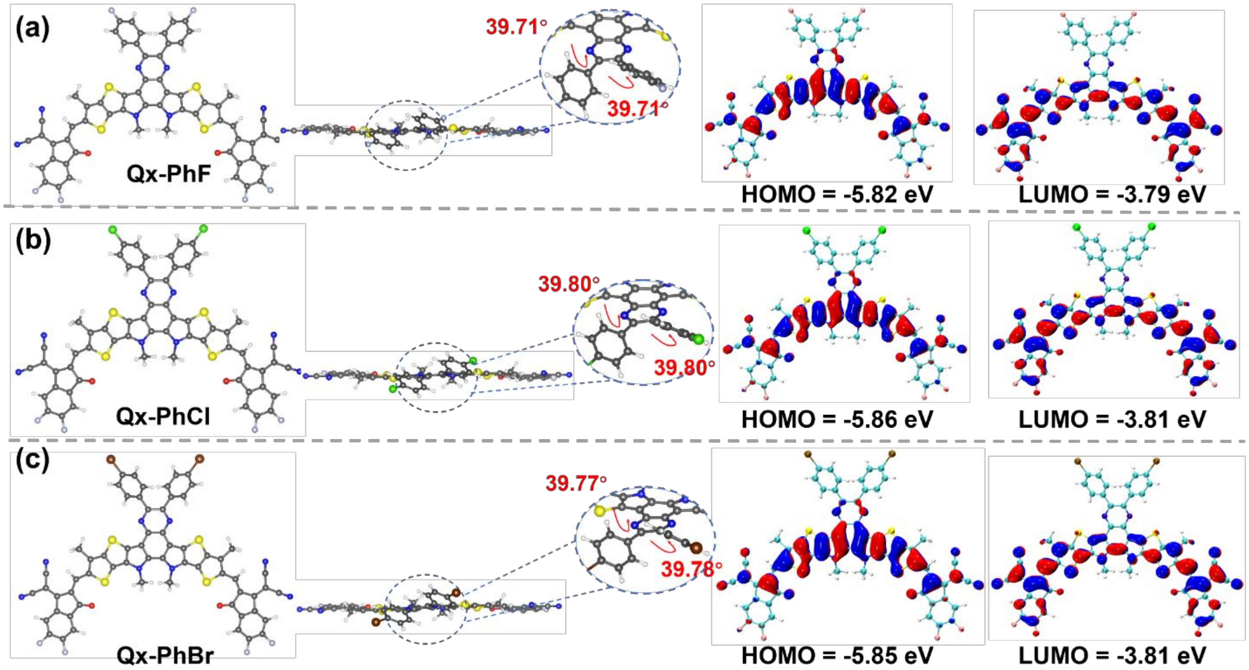

Figure S1. DFT derived molecular structures and HOMOs/LUMOs of **Qx-PhF** (a), **Qx-PhCl (**b), and **Qx-PhBr** (c).

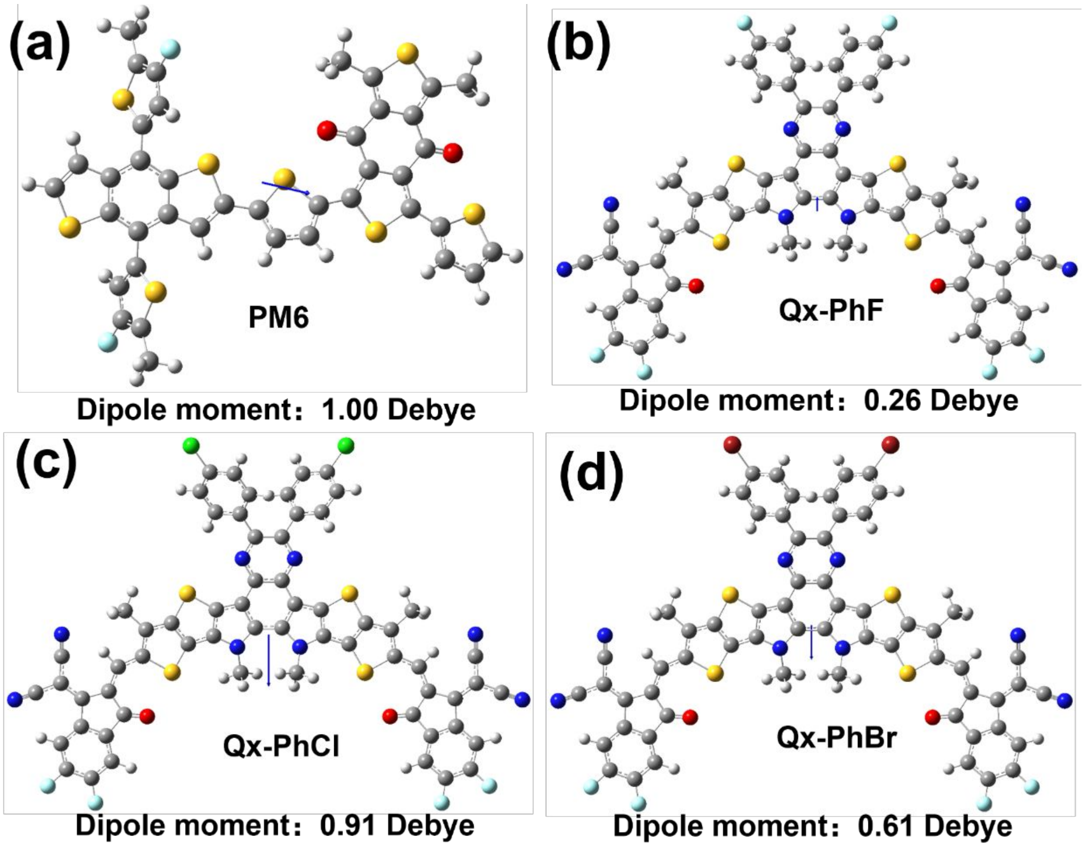

Figure S2. Computed molecular configurations and dipole moments of PM6-monomer (a), **Qx-PhF** (b), **Qx-PhCl** (c), and **Qx-PhBr** (d).

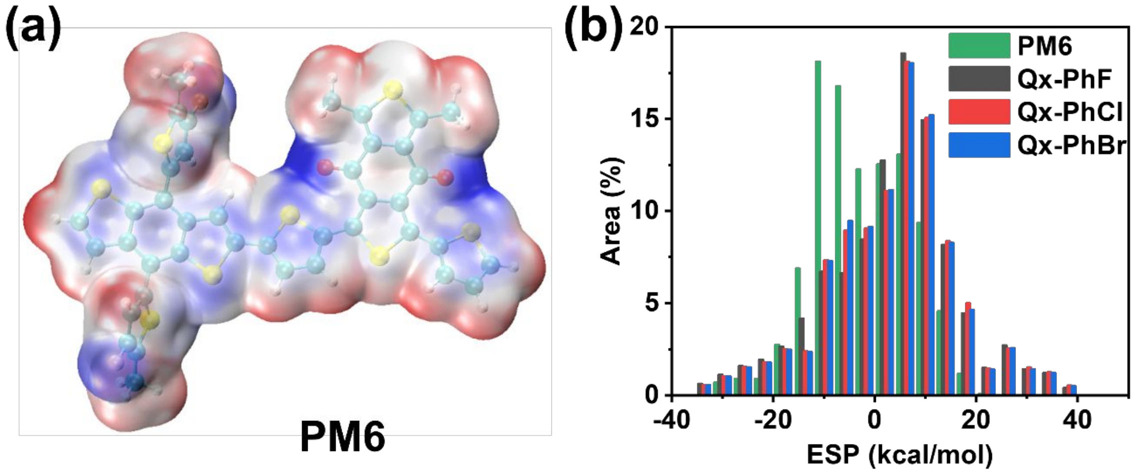

Figure S3. Computed ESP of PM6-monomer (a), and the corresponding ESP distributions (b).

Table S1 Summary of quantified ESP area distributions and overall average ESP values of **Qx-PhF, Qx-PhCl** and **Qx-PhBr** (Isosurface = 0.001 a.u.).

NFAs	Overall surface area [Å^2]	Negative ESP surface area [%]	MPI [kcal/mol]	Minimal value [kcal/mol]	Maximal value [kcal/mol]	Overall average value [kcal/mol]
**Qx-F**	982.17	29.00	11.19	-33.30	40.55	5.15
**Qx-Cl**	1012.36	30.46	11.11	-33.01	41.22	5.42
**Qx-Br**	1022.99	31.19	10.99	-33.06	41.11	5.28
PM6	723.10	53.81	7.66	-29.49	22.67	-0.32

2. Synthesis

Materials: Solvents and other commercially available reagents were ordered from Adamas or Energy Chemical and were used without further purification unless otherwise stated. 1,2-dichloroethane (DCE), and chloroform were freshly distilled from appropriate drying agents before use. Column chromatography was performed with the use of silica gel (200-300 mesh). Compound **1** was synthesized according to the reported procedure.[1]

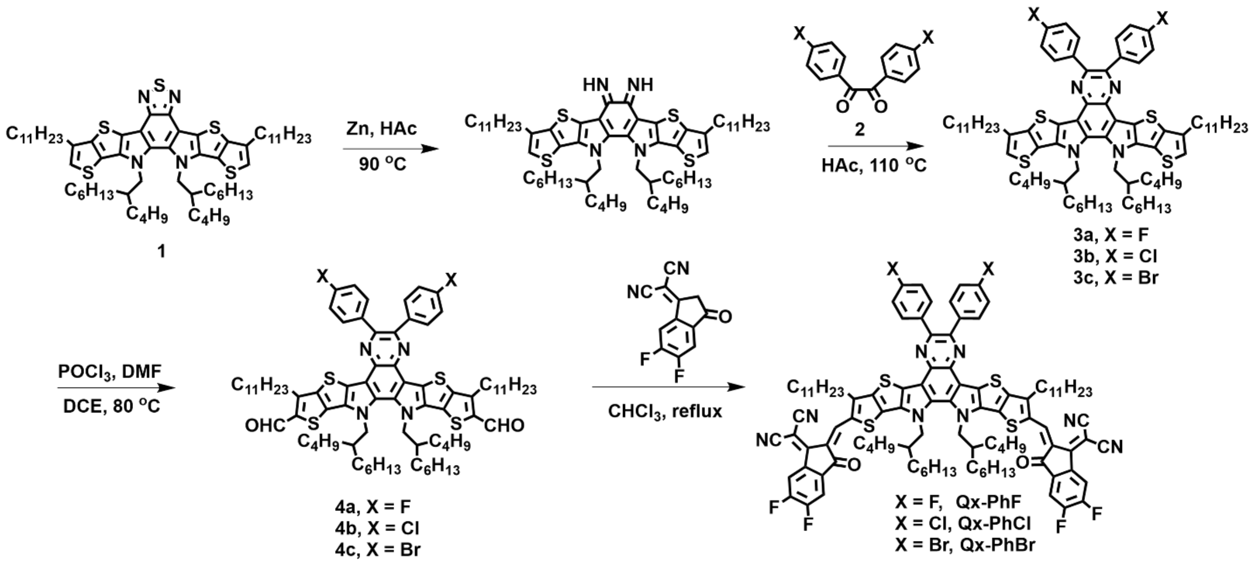

Scheme 1 Synthetic route to the target molecule.

Synthesis of Compound 3a, 3b, 3c[2]: To a solution of compound **1**(1.00 g, 0.92 mmol) in acetic acid (20 mL) was added zinc powder (2.40 g, 36.91 mmol) in one portion. The resulting mixture was stirred at 90°C for 6 h. Subsequently, 20 mL ethyl acetate (EA) was added to the mixture after the solution was cooled to room temperature, and the undissolved solid was removed through filtration. Following the removal of EA via a rotary evaporator, compound **2** (4 eq., 3.69 mmol) was added to the residue. The resulting mixture was then subjected to heating at 110 °C for 12 h. After cooling to room temperature, 100 mL of water was added to the mixture. The organic compounds were extracted using methylene chloride three times. The solvent was then removed under reduced pressure. The resulting crude product was subsequently purified through column chromatography on silica gel, affording compound **3** as a yellow solid.

Compound **3a** (yield: 61%): ^{1}H NMR (400 MHz, $CDCl_3$) δ 7.79-7.76 (m, 4H, ArH), 7.15-7.11 (m, 4H, ArH), 7.01 (s, 2H, ArH), 4.66 (d, J = 7.2 Hz, 4H, -CH_2-), 2.85 (t, J = 7.6 Hz, 4H, -CH_2-), 2.18

– 2.10 (m, 2H,-CH-), 1.90 – 1.83 (m, 4H, $-CH_2-$), 1.47-1.27 (m, 34H, $-CH_2-$), 1.11 – 0.80 (m, 30H, $-CH_2-$), 0.72 – 0.65 (m, 12H, $-CH_3$).

Compound **3b** (yield: 58%): ^{1}H NMR (400 MHz, $CDCl_3$) δ 7.73 (d, *J* = 8.4 Hz, 4H, ArH), 7.42 (d, *J* = 8.4 Hz, 4H, ArH), 7.01 (s, 2H, ArH), 4.66 (d, *J* = 7.6 Hz, 4H, $-CH_2-$), 2.85 (t, *J* = 7.6 Hz, 4H, $-CH_2-$), 2.16 – 2.09 (m, 2H,-CH-), 1.90 – 1.82 (m, 4H, $-CH_2-$), 1.47-1.22 (m, 34H, $-CH_2-$), 1.10 – 0.80 (m, 30H, $-CH_2-$), 0.67 – 0.58 (m, 12H, $-CH_3$).

Compound **3c**(yield: 62%): ^{1}H NMR (400 MHz, $CDCl_3$) δ 7.68 (d, *J* = 8.4 Hz, 4H, ArH), 7.57 (d, *J* = 8.4 Hz, 4H, ArH), 7.01 (s, 2H, ArH), 4.65 (d, *J* = 7.6 Hz, 4H, $-CH_2-$), 2.85 (t, *J* = 7.6 Hz, 4H, $-CH_2-$), 2.17 – 2.09 (m, 2H,-CH-), 1.90 – 1.83 (m, 4H, $-CH_2-$), 1.47-1.27 (m, 34H, $-CH_2-$), 1.13 – 0.80 (m, 30H, $-CH_2-$), 0.72 – 0.65 (m, 12H, $-CH_3$).

Synthesis of compounds 4a, 4b, 4c: Compounds **3** (0.237 mmol) were dissolved in 1,2-dichloroethane (30 ml) in a three-neck flask. $POCl_3$ (1.0 mL) and DMF (1.0 mL) were added to the solution, and the resulting solution was stirred at 80 °C overnight under nitrogen. After that, the mixture was quenched with ice water (50 mL) and neutralized with aqueous AcONa. Then the mixture was extracted with dichloromethane three times. The solvent was removed under reduced pressure. The crude product was subsequently isolated by column chromatography on silica gel to afford compound **4** as an orange solid.

Compound **4a** (yield: 90 %): ^{1}H NMR (400 MHz, $CDCl_3$) δ 10.2 (s, 2H, -CHO), 7.78-7.75 (m, 4H, ArH), 7.17-7.13 (d, *J* = 8.4 Hz, 4H, ArH), 4.68 (d, *J* = 8.0 Hz, 4H, $-CH_2-$), 3.23 (t, *J* = 7.6 Hz, 4H, $-CH_2-$), 2.18 – 2.09 (m, 2H,-CH-), 1.97 – 1.89 (m, 4H, $-CH_2-$), 1.54 - 1.25 (m, 34H, $-CH_2-$), 1.12 – 0.80 (m, 30H, $-CH_2-$), 0.68 - 0.60 (m, 12H, $-CH_3$).

Compound **4b** (yield: 90%): ^{1}H NMR (400 MHz, $CDCl_3$) δ 10.1 (s, 2H, -CHO), 7.77 (d, *J* = 8.0 Hz, 4H, ArH), 7.44 (d, *J* = 8.0 Hz, 4H, ArH), 4.69 (d, *J* = 7.6 Hz, 4H, $-CH_2-$), 3.22 (t, *J* = 7.6 Hz, 4H, $-CH_2-$), 2.14 – 2.10 (m, 2H,-CH-), 1.97 – 1.89 (m, 4H, $-CH_2-$), 1.50 - 1.26 (m, 34H, $-CH_2-$), 1.12 – 0.80 (m, 30H, $-CH_2-$), 0.71 - 0.60 (m, 12H, $-CH_3$).

Compound **4c** (yield: 92%): ^{1}H NMR (400 MHz, $CDCl_3$) δ 10.1 (s, 2H, -CHO), 7.67 (d, *J* = 8.4 Hz, 4H, ArH), 7.60 (d, *J* = 8.4 Hz, 4H, ArH), 4.68 (d, *J* = 7.6 Hz, 4H, $-CH_2-$), 3.22 (t, *J* = 7.6 Hz,

4H, $-CH_2-$), 2.12 – 2.09 (m, 2H,-CH-), 1.97 – 1.89 (m, 4H, $-CH_2-$), 1.50 - 1.26 (m, 34H, $-CH_2-$), 1.12 – 0.80 (m, 30H, $-CH_2-$), 0.68 - 0.60 (m, 12H, $-CH_3$).

Synthesis of compounds Qx-PhF, Qx-PhCl, Qx-PhBr: Compound **4** (0.0693 mmol, 1 eq.) and 2-(5,6-difluoro-3-oxo-2,3-dihydro-1H-inden-1-ylidene)malononitrile (0.20 mmol, 3 eq) were dissolved in chloroform (20 mL) in a three-neck flask. The resulting solution was flushed with nitrogen for 20 min. Then 1.0 mL of pyridine was added to the reaction mixture, which was then stirred at 65 °C overnight under a nitrogen atmosphere. After completion of the reaction, the reaction mixture was poured into methanol, and the resulting solid was collected by filtration. The crude product was purified by column chromatography on silica gel, affording a black solid.

Qx-PhF (yield: 80%). ^1H NMR (600 MHz, $CDCl_3$, ppm) δ 9.14 (s, 2H, ArH), 8.56-8.53 (m, 2H, ArH), 7.77-7.75 (m, 4H, ArH), 7.71-7.68 (m, 2H, ArH), 7.19-7.16 (m, 4H, ArH), 4.86-4.78 (m, 4H, $-CH_2-$), 3.25 (t, 4H, J = 7.8 Hz, $-CH_2-$), 2.22-2.20 (m, 2H, -CH-), 1.88-1.85 (m, 4H, $-CH_2-$), 1.54-1.49 (m, 4H, $-CH_2-$), 1.39-1.35 (m, 4H, $-CH_2-$), 1.31-0.89 (m, 64H, $-CH_2-$), 0.73 - 0.63 (m, 12H, $-CH_3$).

^{13}C NMR (150 MHz, $CDCl_3$, ppm) δ 186.28, 162.62, 159.02, 154.25, 149.83, 146.46, 138.40, 136.81, 136.36, 135.46, 134.63, 133.55, 133.37, 132.27, 132.22, 130.90, 119.83, 119.60, 115.84, 115.69, 115.18, 114.79, 68.53, 55.78, 39.33, 32.05, 31.75, 31.59, 30.58, 29.88, 29.84, 29.77, 29.66, 29.64, 29.48, 28.21, 28.08, 25.65, 25.51, 23.05, 23.02, 22.81, 22.63, 14.23, 14.17, 13.67, 13.95.

MS (MALDI-TOF): m/z, Calc. for $[C_{104}H_{110}F_6N_8O_2S_4]^+$ 1745.75, found: 1745.36.

Qx-PhCl (yield: 80%).^1H NMR (600 MHz, $CDCl_3$, ppm) δ 9.12 (s, 2H, ArH), 8.54-8.51 (m, 2H, ArH), 7.72- 7.68 (m, 6H, ArH), 7.46 (d, 2H, J = 6.0 Hz, ArH), 4.85-4.81 (m, 4H, $-CH_2-$), 3.24 (t, 4H, J = 8.4 Hz, $-CH_2-$), 2.22-2.20 (m, 2H, -CH-), 1.89-1.84 (m, 4H, $-CH_2-$), 1.54-1.49 (m, 4H, $-CH_2-$), 1.39-1.35 (m, 4H, $-CH_2-$), 1.31-0.84 (m, 64H, $-CH_2-$), 0.73 - 0.62 (m, 12H, $-CH_3$).

^{13}C NMR (150 MHz, $CDCl_3$, ppm) δ 186.27, 158.95, 155.36, 154.22, 149.61, 146.47, 138.40, 137.69, 136.30, 135.45, 134.74, 133.59, 133.37, 131.67, 130.82, 128.98, 119.85, 119.53, 115.15, 114.99, 114.76, 112.54, 112.42, 68.54, 55.80, 39.35, 32.05, 31.75, 31.59, 30.59, 29.87, 29.84, 29.77, 29.66, 29.61, 29.48, 28.22, 28.10, 25.67, 25.54, 23.05, 23.02, 22.82, 22.63, 14.24, 14.17, 13.97, 13.95.

MS (MALDI-TOF): m/z, Calc. for $[C_{104}H_{111}Cl_2F_4N_8O_2S_4]^+$ 1778.71, found: 1779.08.

Qx-PhBr (yield: 80%). ^{1}H NMR (600 MHz, $CDCl_3$, ppm) δ 9.14 (s, 2H, ArH), 8.56-8.53 (m, 2H, ArH), 7.71- 7.69 (m, 2H, ArH), 7.65-7.61 (m, 8H,ArH), 4.84-4.81 (m, 4H, $-CH_2-$), 3.25 (t, 4H, J = 8.4 Hz, $-CH_2-$), 2.22-2.20 (m, 2H, -CH-), 1.90-1.84 (m, 4H, $-CH_2-$), 1.54-1.49 (m, 4H, $-CH_2-$), 1.40-1.35 (m, 4H, $-CH_2-$), 1.31-0.84 (m, 64H, $-CH_2-$), 0.73 - 0.63 (m, 12H, $-CH_3$).
^{13}C NMR (150 MHz, $CDCl_3$, ppm) δ 186.28, 158.99, 154.23, 149.60, 146.47, 138.29, 136.29, 135.46, 134.79, 133.61, 133.38, 131.94, 130.86, 123.81, 119.88, 119.51, 115.16, 115.01, 114.76, 112.44, 68.58, 55.79, 39.33, 32.06, 31.74, 31.61, 30.63, 29.88, 29.84, 29.77, 29.66, 29.61, 29.48, 28.20, 28.07, 25.63, 25.50, 23.04, 23.01, 22.82, 22.62, 14.25, 14.17, 13.97, 13.94.
MS (MALDI-TOF): m/z, Calc. for $[C_{104}H_{111}Br_2F_4N_8O_2S_4]^+$ 1868.60, found: 1868.92.

3. Thermal analyses

Thermogravimetric analysis (TGA) and differential scanning calorimetry (DSC) were conducted on a METTLER TOLEDO TGA/DSC 1 thermogravimetric analyzer with thermal balance under protection of nitrogen at a heating rate of 10 °C min^{-1}.

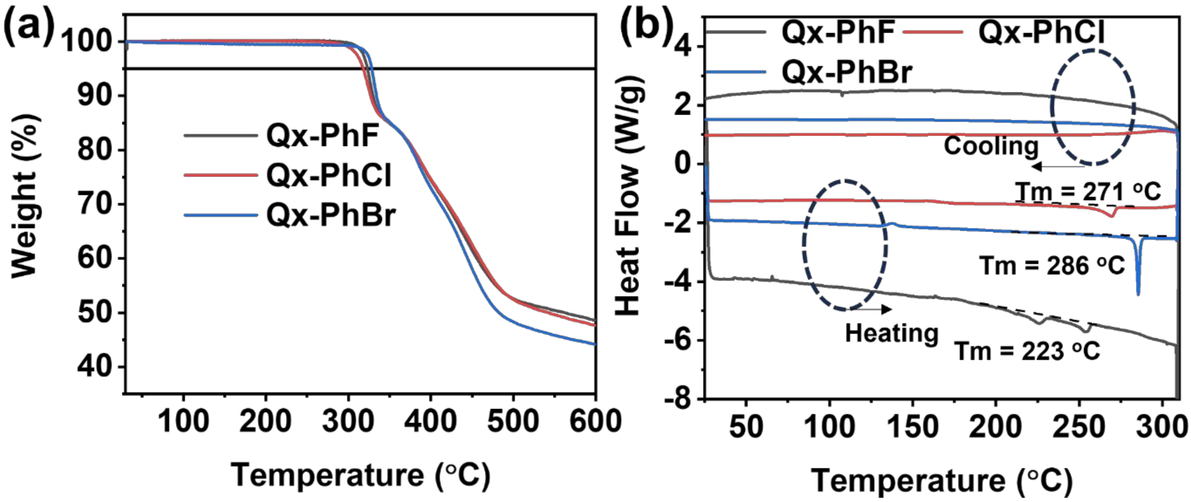

Figure S4. (a) TGA plots of **Qx-PhF**, **Qx-PhCl**, and **Qx-PhBr** with a heating rate of 10 °C min^{-1} under N_2 atmosphere. (b) DSC plots of **Qx-PhF**, **Qx-PhCl**, and **Qx-PhBr** with a heating/cooling rate of 10 °C min^{-1} under N_2 atmosphere.

4. Photophysical property characterizations

Ultraviolet−visible (UV-vis) absorption spectra were recorded with a Perkin Elmer Lambda 365 spectrophotometer from the dilute chloroform solution and as-casted thin films.

5. Electrochemistry property characterizations

Cyclic voltammetry (CV) was performed by CHI600E electrochemical workstation, applying a three-electrode system with a platinum wire electrode as a counter electrode, Ag/AgCl electrode as a reference electrode, and a glassy carbon electrode as a working electrode. The electrolyte was prepared by dissolving tetrabutylammonium hexafluorophosphate (n-Bu_4PF_6) acetonitrile (0.1 M). The CV curves versus the potential of the reference electrode were recorded, which was calibrated by the ferrocene–ferrocenium (Fc/Fc^+) redox couple. HOMO and LUMO energies were estimated from the onset oxidation and reduction potentials (*EonsetNFAr red/oxi*), respectively, using equation (1):

$$E_{HOMO/LUMO} =- (e(E\text{onset, NFA red/oxi}-E\text{onset, Fc red/oxi}) + 4.8\text{ eV}] \;\ldots\ldots\; \text{eq. 1}$$

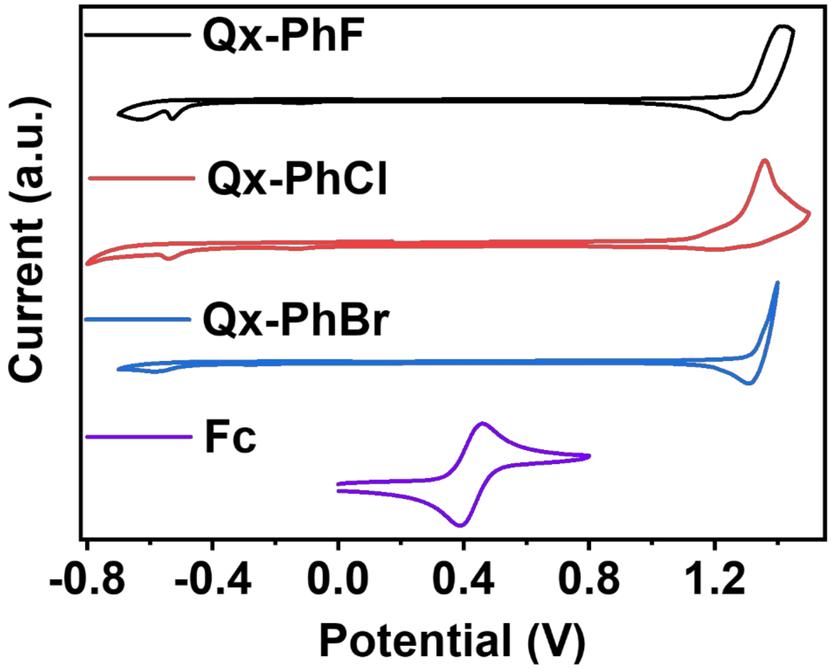

Figure S5. CV curves for the indicated compounds.

6. Fabrication of organic solar cells

The device structure for binary OSCs was ITO/PEDOT:PSS/PM6:NFAs/PNDIT-F3N/Ag in this study. ITO-coated glass substrates (15 Ω sq^{-1}) were sequentially cleaned using detergent water, deionized water, acetone, and isopropyl alcohol in an ultrasonic bath for 30 min each, and followed by UV exposure for 30 min in a UV-ozone chamber. A thin layer (ca. 30 nm) of PEDOT:PSS (Bayer Baytron 4083) was first spin-coated onto the pre-cleaned ITO-coated glass substrates at 4000 rpm and baked at 120 °C for 15 min under ambient conditions. The substrates were then transferred to a nitrogen-filled glove box for active layer preparation. The donor and acceptor materials were dissolved in chloroform (CF) with a total concentration of 14.5 mg/mL (donor: acceptor = 1:1.2 w/w) and stirred at 50 ℃ for 60 minutes before active layer processing. 1-Chloronaphthalene (CN) was added to the precursors at a volume fraction of 0.3%, 0.4%, or 0.5%. The blend solution was spin-coated at 2200 rpm for 30 s to form a thin film on the substrate, followed by thermal annealing at 90 ℃, 100 ℃, or 110 ℃ for 10 min. The active layer thickness was approximately 100 nm. Next, PNDIT-F3N (0.5 mg mL^{-1}, in methanol with 0.5% v/v acetic acid) was spin-coated on the active layer at 1800 rpm for 30 s as the electron transporting layer. Finally, the substrates were transferred to a thermal evaporator, and Ag electrode was evaporated at a base pressure of 2×10^{-5} Pa. The *J–V* characteristics were assessed under AM 1.5 G illumination conditions (100 mW cm^{-2}) (Enli Technology Co., Ltd. SS-X50R). The *J–V* data were recorded using a Keithley 2400 source-measure unit. The active area of each cell is 0.1 cm^{-2}. The external quantum efficiency (EQE) analysis was conducted utilizing certified IPCE equipment (Zolix Instruments, Inc, Solar Cell Scan 100).

Table S2 Device optimizations for **PM6:Qx-PhF** OSCs.

D/A	CN	TA (℃)	V_{oc}(V)	J_{sc}(mA/cm^2)	FF (%)	PCE (%)
1:1.1	0.4%	100	0.88	21.99	72.81	14.10
1:1.2	0.4%	100	0.88	22.67	73.02	14.57
1:1.3	0.4%	100	0.88	22.23	72.96	14.28
	0.3%	100	0.88	21.72	73.20	14.04
1:1.2	0.4%	100	0.88	22.62	73.51	14.66
	0.5%	100	0.88	21.96	73.22	14.13
	0.4%	90	0.88	22.56	72.47	14.46
1:1.2	0.4%	100	0.88	22.56	74.22	14.77
	0.4%	110	0.88	21.64	74.00	14.13

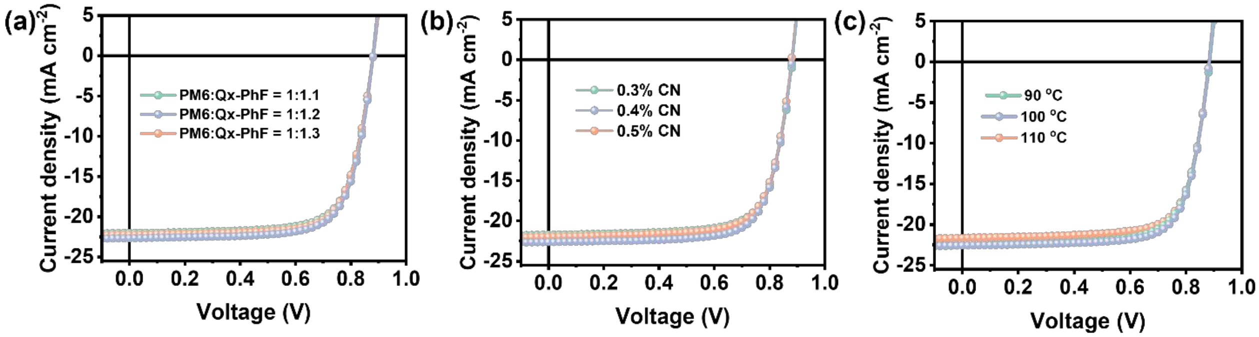

Figure S6. *J-V* curves OSCs optimization for **PM6:Qx-PhF** based devices, (a) D:A ratio optimizations, (b) volume of additives optimization with D:A = 1.1.2, (c) thermal annealing temperature optimization for the devices with D : A = 1 : 1.2 and 0.4% CN.

Table S3 Device optimizations for **PM6:Qx-PhCl** OSCs.

D/A	CN	TA (℃)	V_{oc}(V)	J_{sc}(mA/cm^2)	FF (%)	PCE (%)
1:1	0.4%	100	0.89	20.90	68.59	12.68
1:2	0.4%	100	0.89	21.33	71.07	13.42
1:3	0.4%	100	0.89	20.79	70.96	13.07
1:2	0.3%	100	0.89	20.96	69.40	12.91
	0.4%	100	0.89	21.31	71.62	13.55
	0.5%	100	0.88	20.65	71.26	13.01
1:2	0.4%	90	0.88	21.43	69.74	13.21
	0.4%	100	0.89	21.49	71.21	13.59
	0.4%	110	0.88	21.48	69.09	13.11

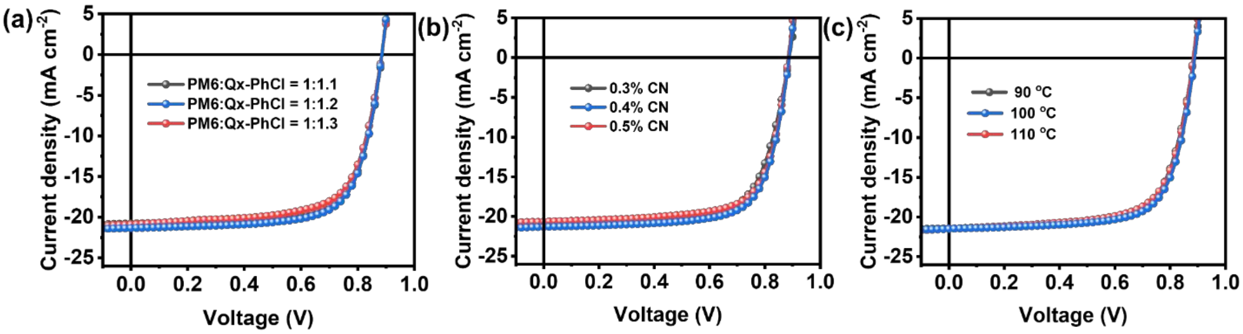

Figure S7. *J-V* curves OSCs optimization for **PM6:Qx-PhCl** based devices, (a) D:A ratio optimizations, (b) volume of additives optimization with D:A = 1 : 1.2, (c) thermal annealing temperature optimization for the devices with D : A = 1 : 1.2 and 0.4% CN.

Table S4 Device optimization for **PM6:Qx-PhBr** OSCs.

D/A	CN	TA (℃)	V_{oc}(V)	J_{sc}(mA/cm^2)	FF (%)	PCE (%)
1:1.1	0.4%	100	0.90	25.05	75.05	16.98
1:1.2	0.4%	100	0.91	25.22	75.34	17.23
1:1.3	0.4%	100	0.91	24.95	75.30	17.03
	0.3%	100	0.91	25.07	75.09	17.05
1:1.2	0.4%	100	0.90	25.67	75.56	17.45
	0.5%	100	0.90	25.26	74.78	16.92
	0.4%	90	0.91	24.43	74.54	16.53
1:1.2	0.4%	100	0.91	25.59	75.53	17.58
	0.4%	110	0.91	25.11	75.23	17.16

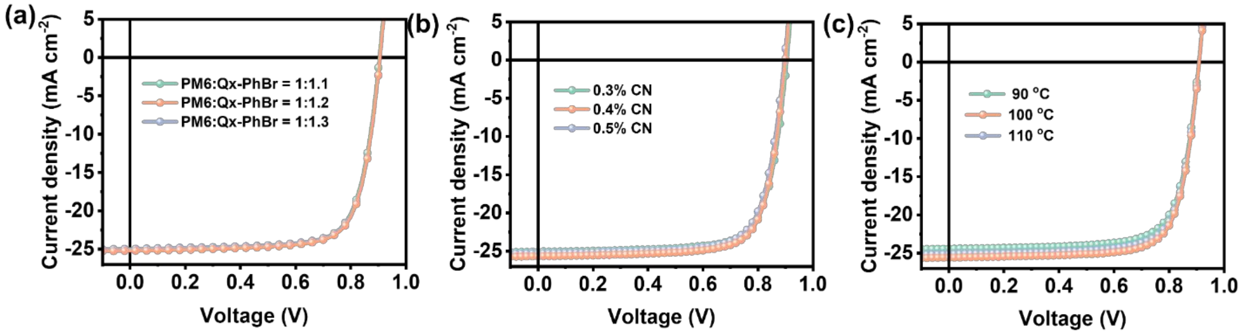

Figure S8. *J-V* curves OSCs optimization for **PM6:Qx-PhF** based devices, (a) D:A ratio optimizations, (b) volume of additives optimization with D:A = 1.1.2, (c) thermal annealing temperature optimization for the devices with D : A = 1 : 1.2 and 0.4% CN.

7. Stability

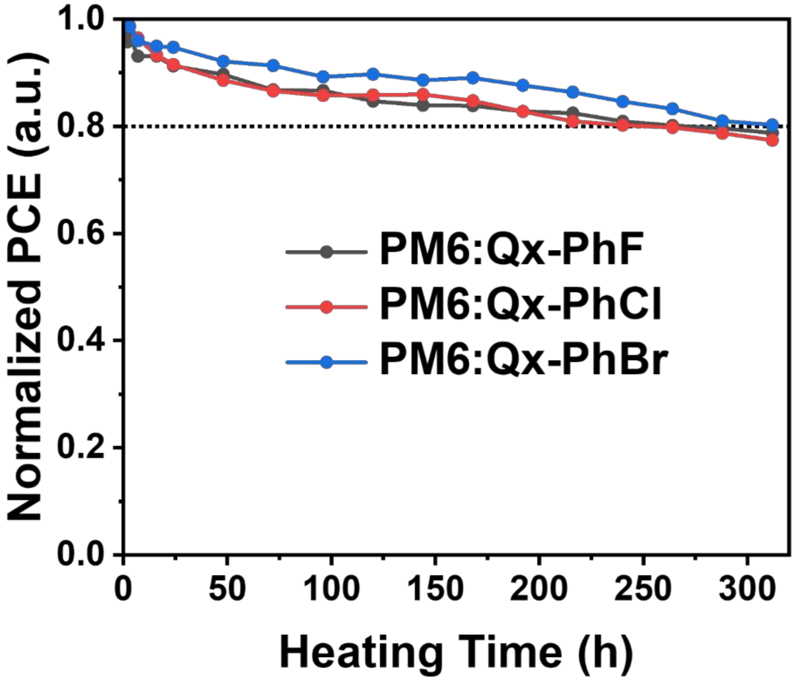

Figure S9. The PCE vs heating time of the indicated devices.

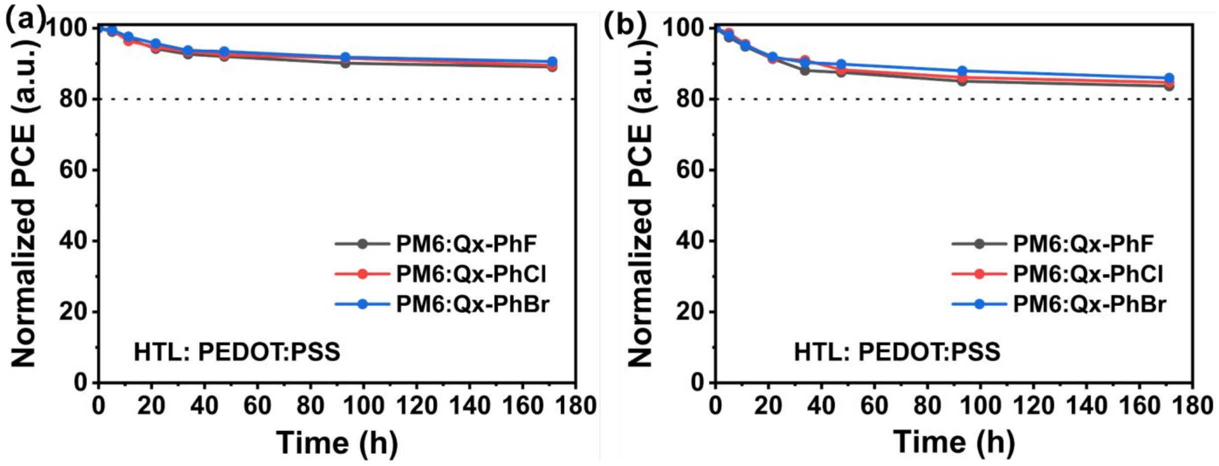

Figure S10. Normalized PCEs of the indicated binary devices stored at (a) room temperature, (b) under simulated sunlight.

8. Mobility measurements

The charge mobility (hole and electron mobility) of the photoactive layer was determined through the utilization of the space-charge-limited current (SCLC) model, by analyzing the dark current characteristics of hole and electron-only diodes. Hole-only diode configuration: Glass/ITO/PEDOT:PSS/active layer/MoO_3/Ag, Electron-only diode configuration: Glass/ITO/ZnO/PNDIT-F3N/active layer/ PNDIT-F3N/Ag. For the hole-only device structure, V_{bi} = 0 V (flat band pattern formed by MoO_3-MoO_3); For the electron-only device structure, V_{bi} = 0.5 V. The active layer thickness was checked by a Tencor surface profilometer. The electric-field dependent SCLC mobility was estimated by the following equation:

$$J = \frac{9}{8}\varepsilon_0\varepsilon_r\mu_0 exp\left(0.89\beta\sqrt{\frac{V-V_{bi}}{L}}\right)\frac{(V-V_{bi})^2}{L^3}$$

where J is the current density, L is the photoactive layer thickness, ε_r is the relative dielectric constant, ε_0 is the permittivity of free space (8.85×10^{-12} F m^{-1}), and V is the internal voltage in the device.

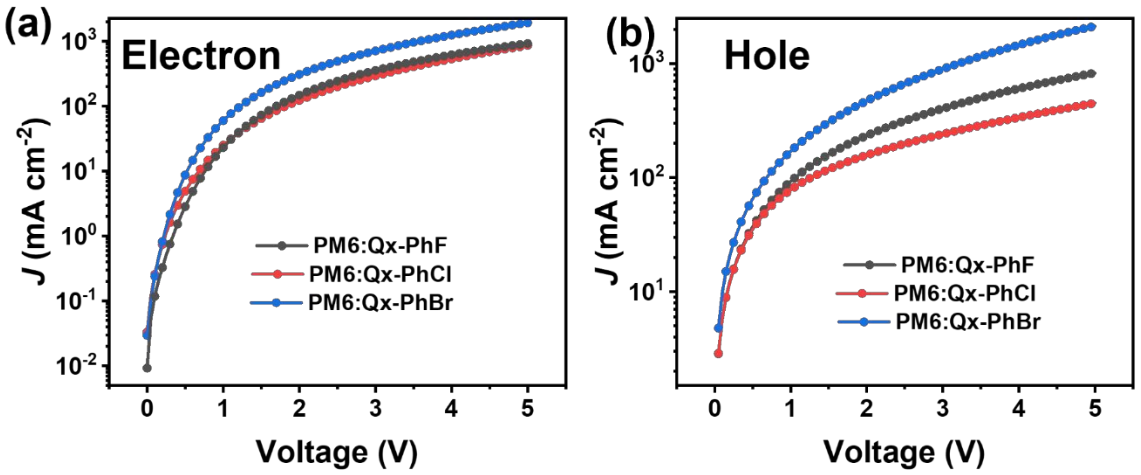

Figure S11. The current density-voltage relationship for electron mobility (a) and hole mobility of **PM6:Qx-PhF, PM6:Qx-PhCl** and **PM6:Qx-PhBr**.

Table S5 The calculated charge mobility for the indicated blend.

Blend	μ_e (cm^2 V^{-1} s^{-1})	μ_h (cm^2 V^{-1} s^{-1})	μ_e/μ_h
**PM6:Qx-PhF**	2.18×10^{-4}	5.08×10^{-4}	0.43
**PM6:Qx-PhCl**	1.31×10^{-4}	4.62×10^{-4}	0.28
**PM6:Qx-PhBr**	4.10×10^{-4}	6.51×10^{-4}	0.63

9. Transient photovoltage (TPV) and transient photocurrent (TPC)

The TPV measurement was carried out under standard solar irradiance conditions by illuminating the device with a white light-emitting diode, while maintaining the device in an open-circuit state. The TPC evaluation was performed under dark conditionwith the device set to a short-circuit state. The output signal was recorded by Keysight oscilloscope. The photovoltage decay kinetics of all devices follow a mono-exponential decay: $\delta V = Aexp(-t/\tau)$ where t is the time, and τ is the charge carrier lifetime.

10. AFM and TEM measurements

Atomic force microscopy (AFM) measurements were recorded in the tapping mode on Scan AsystTM. Transmission electron microscope (TEM) measurements were performed with a FEI Tecnai G2 F20 electron microscopy.

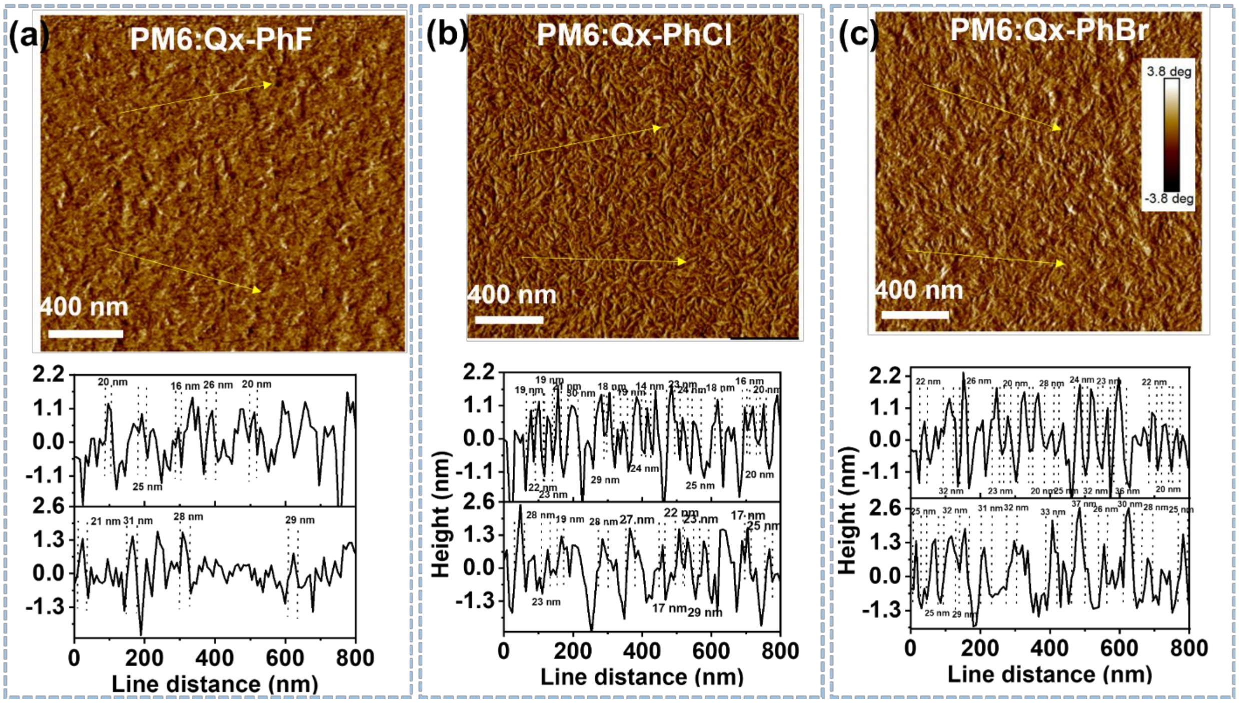

Figure S12. AFM height images of **PM6**:**Qx-PhF** (a), **PM6**:**Qx-PhCl** (b), and **PM6**:**Qx-Br** (c), and corresponding two line-cut profiles.

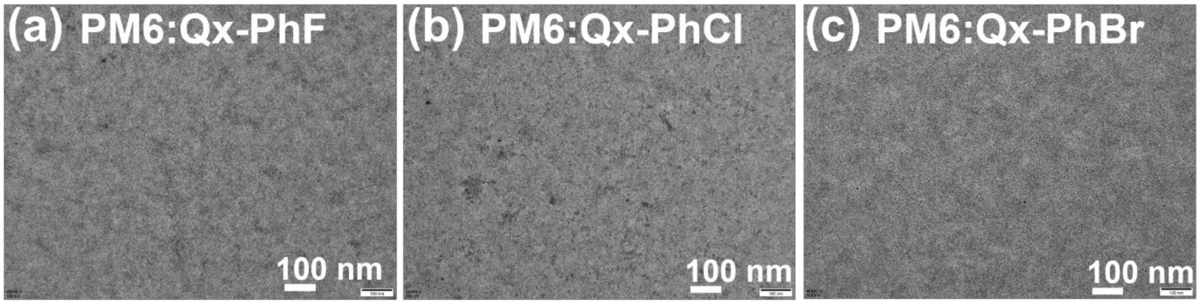

Figure S13. TEM images of **PM6:Qx-PhF**, **PM6:Qx-PhCl** and **PM6:Qx-PhBr**.

11.GIWAXS measurements

GIWAXS measurements were carried out with a Xeuss 2.0 SAXS laboratory beamline using a Cu X-ray source (8.05 keV, 1.54 Å) and a Pilatus3R 300K detector. The incidence angle is 0.2°.

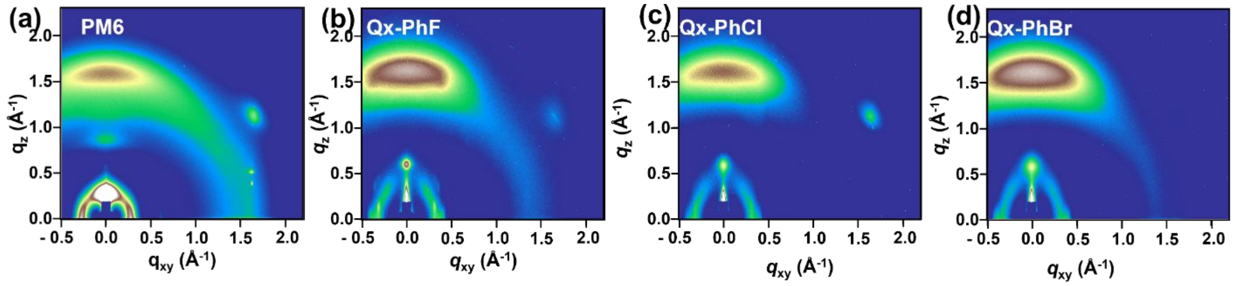

Figure S14. 2D GIWAXS patterns of pristine **PM6** (a), **Qx-PhF** (b), **Qx-PhCl** (c), **Qx-PhBr** (d).

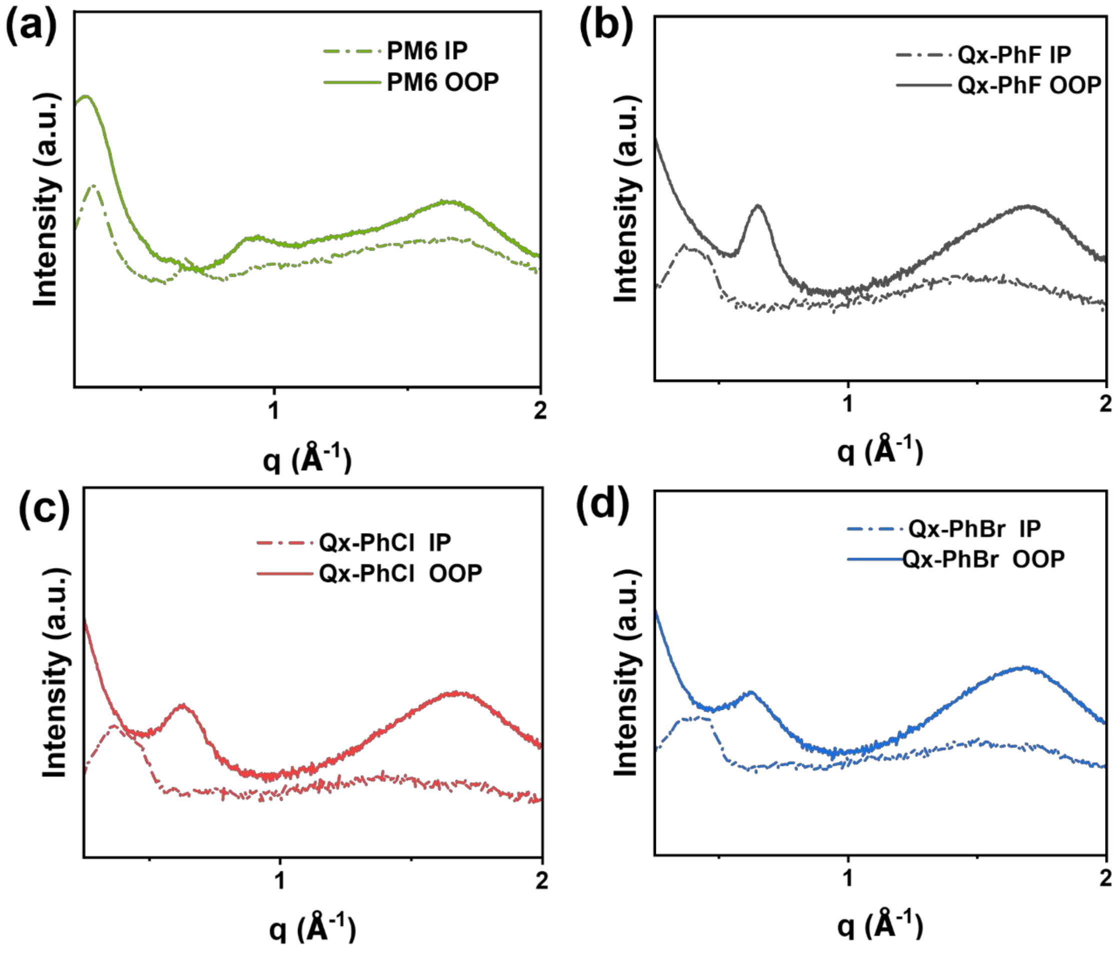

Figure S15. Linecuts for pristine thin films of PM6(a), **Qx-PhF**(b), **Qx-PhCl** (c), **Qx-PhBr** (d).

Table S6. Summary of GIWAXS analysis data for **PM6**, **Qx-PhF**, **Qx-PhCl**, **Qx-PhBr films** in the in-plane direction.

Film	In-plane (100)		
	q (Å^{-1})	d-spacing (Å)	CCL (Å)
**PM6**	0.319	19.69	82.2
**Qx-PhF**	0.367	17.11	72.4
**Qx-PhCl**	0.382	16.44	33.6
**Qx-PhBr**	0.382	16.44	42.0

Table S7. Summary of GIWAXS analysis data for **PM6**, **Qx-PhF**, **Qx-PhCl**, **Qx-PhBr** films in the out-of-plane direction.

Film	Out-of-plane (010)		
	q (Å^{-1})	d-spacing (Å)	CCL (Å)
**PM6**	1.641	3.83	22.0
**Qx-PhF**	1.689	3.72	22.0
**Qx-PhCl**	1.670	3.76	21.2
**Qx-PhBr**	1.678	3.74	19.4

Table S8 Summary of GIWAXS analysis data for **PM6:Qx-PhF**, **PM6:Qx-PhCl**, **PM6:Qx-PhBr** films in the in-plane direction.

Film	In-plane (100)		
	q ($Å^{-1}$)	d-spacing (Å)	CCL (Å)
**PM6:Qx-PhF**	0.329	19.10	95.3
**PM6:Qx-PhCl**	0.328	19.15	95.8
**PM6:Qx-PhBr**	0.326	19.26	106.1

Table S9 Summary of GIWAXS analysis data for **PM6:Qx-PhF**, **PM6:Qx-PhCl**, **PM6:Qx-PhBr** films in the out-of-plane direction.

Film	Out-of-plane (010)		
	q ($Å^{-1}$)	d-spacing (Å)	CCL (Å)
**PM6:Qx-PhF**	1.687	3.72	22.7
**PM6:Qx-PhCl**	1.689	3.72	22.7
**PM6:Qx-PhBr**	1.675	3.75	21.6

12. Contact angle measurements

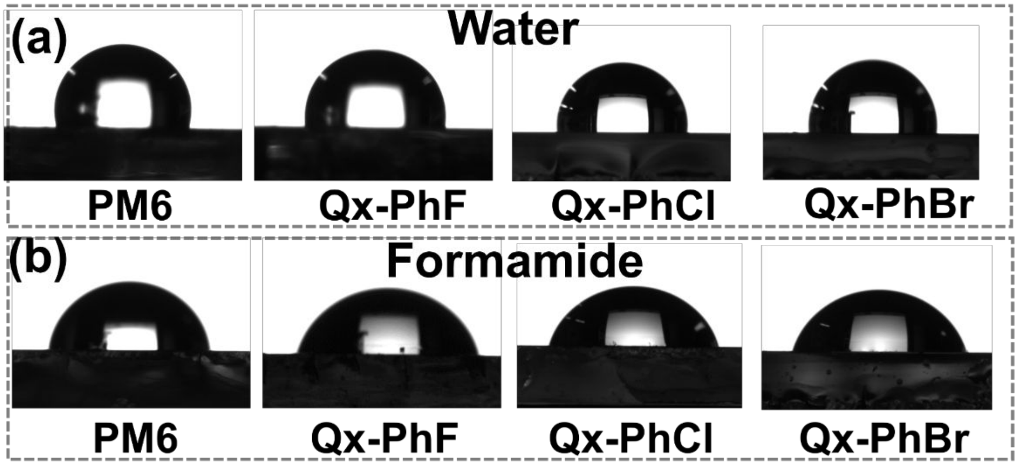

Figure S16. Contact angle with deionized water (a) and formamide (b) drops for **PM6**, **Qx-PhF**, **Qx-PhCl**, **Qx-PhBr** neat films.

Table S10. Summary of contact angles (θ), surface tensions (γ), and Flory–Huggins interaction parameters (χ) for **PM6**, **Qx-PhF**, **Qx-PhCl**, **Qx-PhBr** films.

Material	Water [°]	FA [°]	γ^d [mN m^{-1}]	γ^p [mN m^{-1}]	γ [mN m^{-1}]	$X_{D:A}$ [K]
**PM6**	106.82	84.33	22.28	0.28	22.56	
**Qx-PhF**	102.10	74.78	31.24	0.14	31.38	0.73
**Qx-PhCl**	96.65	74.74	24.90	1.54	26.48	0.16
**Qx-PhBr**	96.36	74.44	25.10	1.59	26.64	0.16

13.Molecular dynamics simulations

All-atom molecular dynamics simulations were performed by Gromacs 2024 program[3]. The GAFF force filed was used. The atomic partial charges were calculated by Gaussian 16 and fitted using the restrained electronic potential (RESP) method by Multiwfn[4]. To simulate the **PM6/Qx-PhF**, **PM6/Qx-PhCl** and **PM6/CH-Qx-PhBr** blend films, whose weight ratios (*w*:*w*) are 1:1.2, a cubic box with length of 18 nm is constructed. To simulate blend films, 18 PM6 hexamer and 83 **Qx-PhF**, 82 **Qx-PhCl** or 78 **Qx-PhBr** molecules are randomly placed into the box by packmol software[5]. Firstly, 2 ns NVT run and 15 ns NPT run are performed at 650 K to compress the system. Then, the system is cooled to 300 K at a rate of 23.3 K/ns, followed by a 10 ns NPT run to equilibrate to system. Lastly, a 5 ns NPT production run is performed at 300 K. The time step is 1.0 fs.

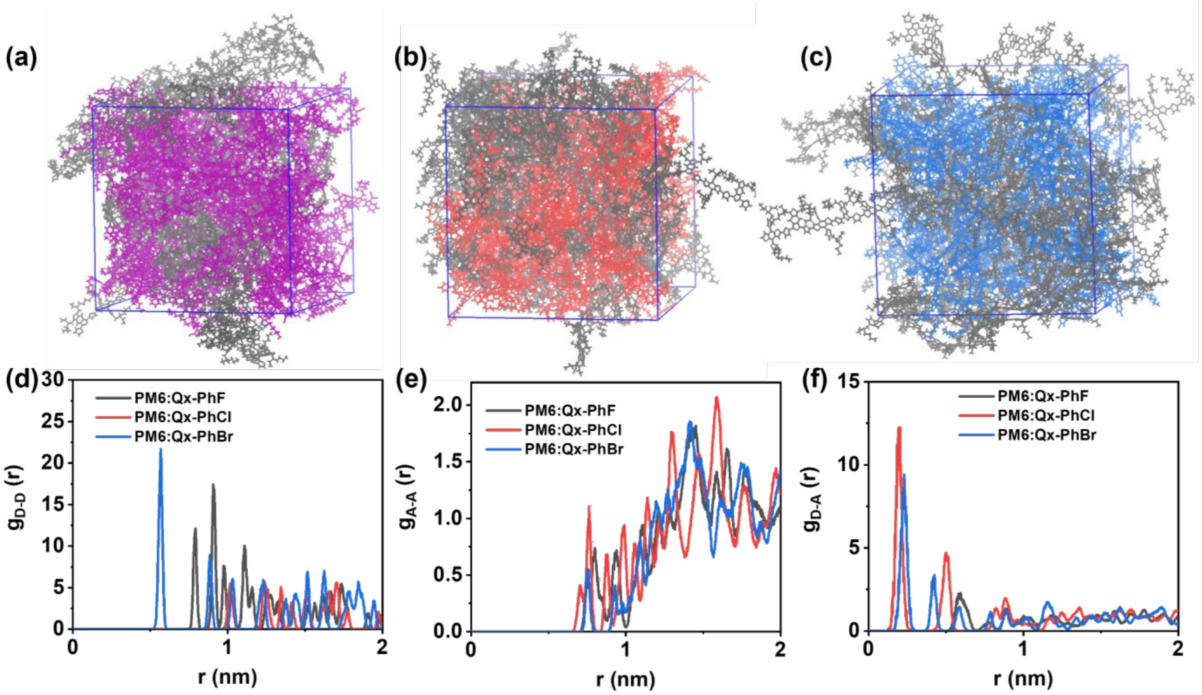

Figure S17 The final snapshots of the **PM6:Qx-PhF** (a), **PM6:Qx-PhCl** (b) and **PM6:Qx-PhBr** (c) blend films by MD simulations. Center-of-mass radial distribution functions g(r) of the PM6 relative to PM6 (d), acceptor relative to acceptor (e), and acceptor relative to PM6 (f) for the simulated blends.

14. Ternary solar cells

The OSCs device structure was ITO/BrDECz/**PM6:BTP-eC9** or **PM6:BTP-eC9:Qx-PhBr**/PDINN/Ag. The BrDECz was synthesized according to our previous study.[6] The optimization of the OSCs was performed following the details in our previous study.[6-7] The BrDECz in ethanol (0.4 mg mL^{-1}) was spin-coated onto precleaned ITO at 3000 rpm for 30 s in air, followed by thermal annealing at 100 °C for 5 min.[6] Next, the active layer was prepared by dissolving a blend of **PM6**, **BTP-eC9**, and **Qx-PhBr** in a weight ratio of 1:1.2:0.05 at a concentration of 16 mg/mL in chloroform, with the addition of 10 mg/mL of 1,4-diiodobenzene as an additive. This solution was spin-coated onto the substrate at 2800 rpm for 30 seconds to form a uniform film, followed by thermal annealing at 90 °C for 5 minutes. Finally, a PDINN layer (0.5 mg/mL in methanol) was spin-coated onto the active layer at 2000 rpm for 30 seconds. The completed devices were then transferred to a vacuum chamber for the deposition of a 100 nm thick Ag electrode via thermal evaporation. The corresponding unencapsulated binary and ternary OSCs were fabricated using PEDOT:PSS and BrDECz as a hole-transporting layer for thermal stability investigation under continuous annealing at 60 °C.

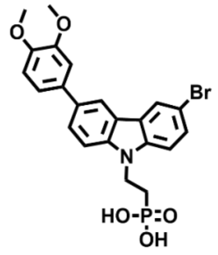

BrDECz

Figure S18. The chemical structure of **BrDECz** in this study.

Table S11 Device metrics for the indicated binary and ternary solar cells**.**

Active layer	V_{OC} (V)	J_{SC} (mA cm^{-2})	*JEQE Cal*[a] (mA cm^{-2})	FF (%)	PCE[b] (%)
**PM6:BTP-eC9**	0.85<br>(0.85 ± 0.002)	28.78<br>(28.42 ± 0.28)	27.57	77.38<br>(77.19 ± 0.40)	19.03<br>(18.72 ± 0.16)
**PM6:BTP-eC9:Qx-PhBr**	0.86<br>(0.86 ± 0.002)	29.40<br>(29.27 ± 0.34)	28.14	79.50<br>(78.08 ± 1.06)	20.14<br>(19.72 ± 0.38)

[a]*JEQE Cal* obtained from the integration of the EQE. [b]Average parameters calculated from 10 devices.

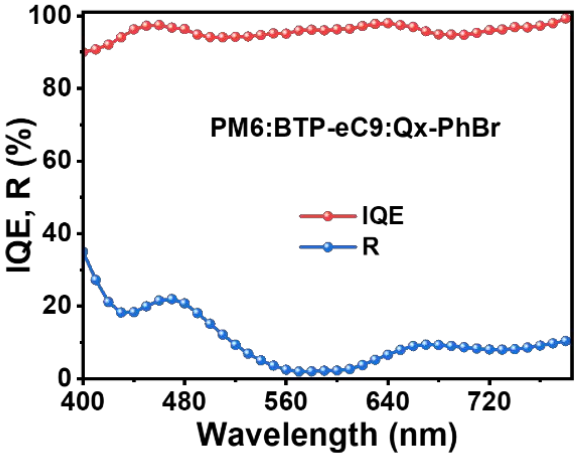

Figure S19 IQE and Reflectance curves of the indicated ternary OSCs.

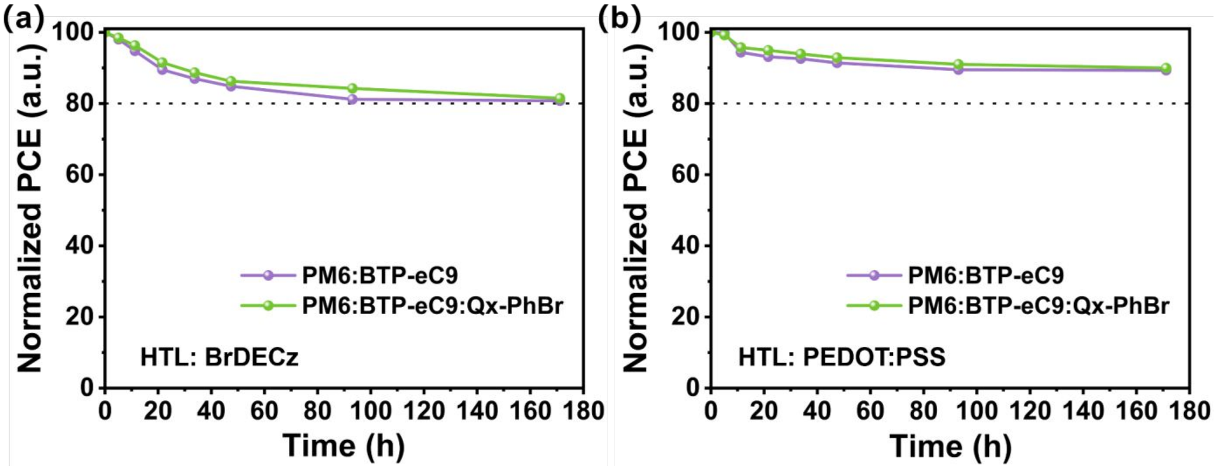

Figure S20 Normalized PCEs of the indicated binary and ternary devices under continuous heating at 80 °C with different hole transporting layers (a) BrDECz, (b) PEDOT:PSS.

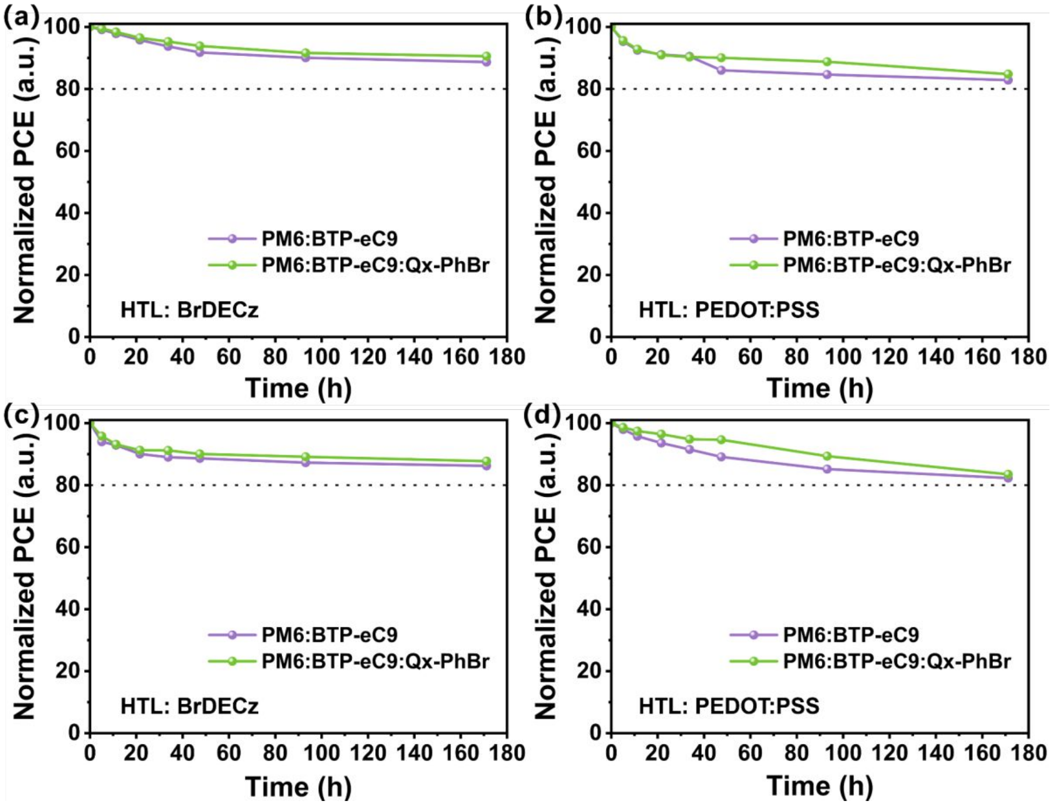

Figure S21 Normalized PCEs of the indicated binary and ternary devices under (a-b) room temperature, and (c-d) simulated sunlight for the indicated HTL-based OSCs.

15. NMR spectra and mass spectroscopic analyses of the indicated materials

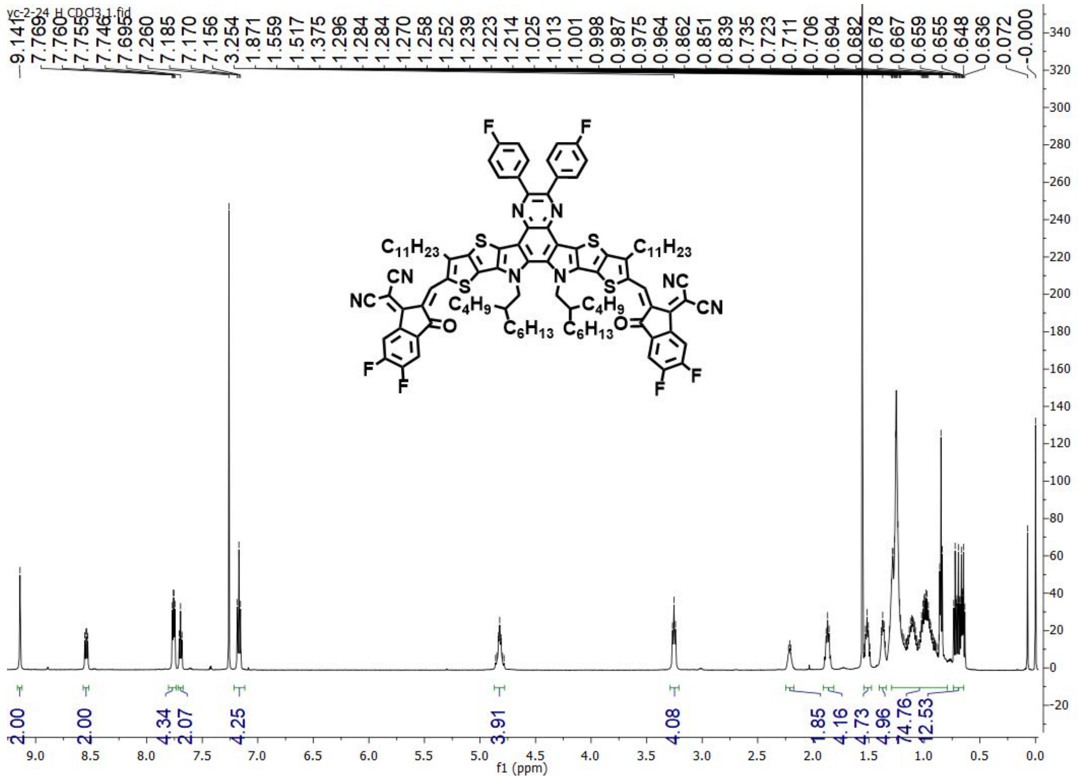

Figure S22 ^{1}H NMR spectrum of **Qx-PhF** in $CDCl_3$.

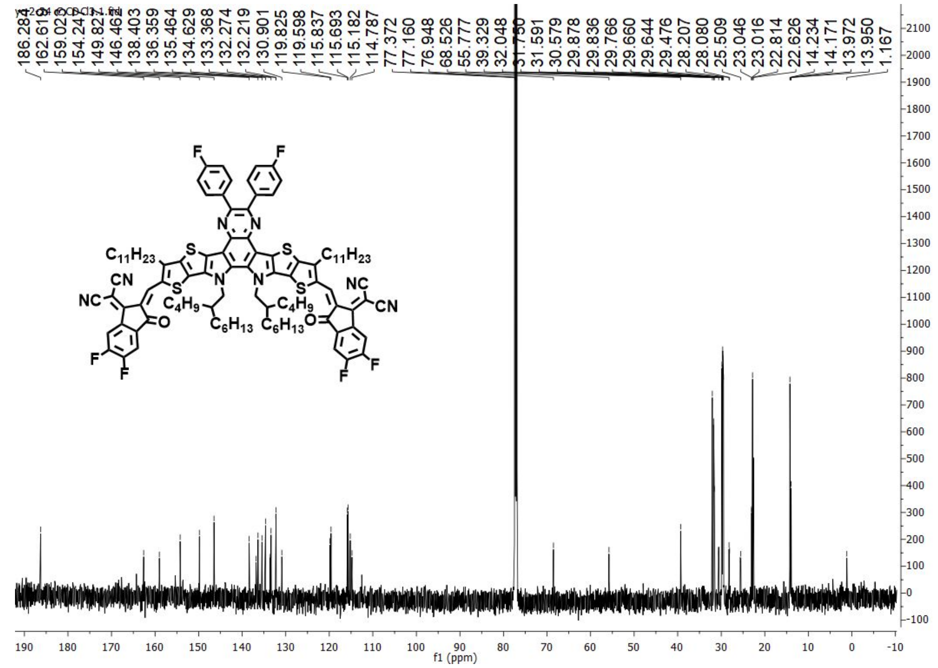

Figure S23 ^{13}C NMR spectrum of **Qx-PhF** in $CDCl_3$.

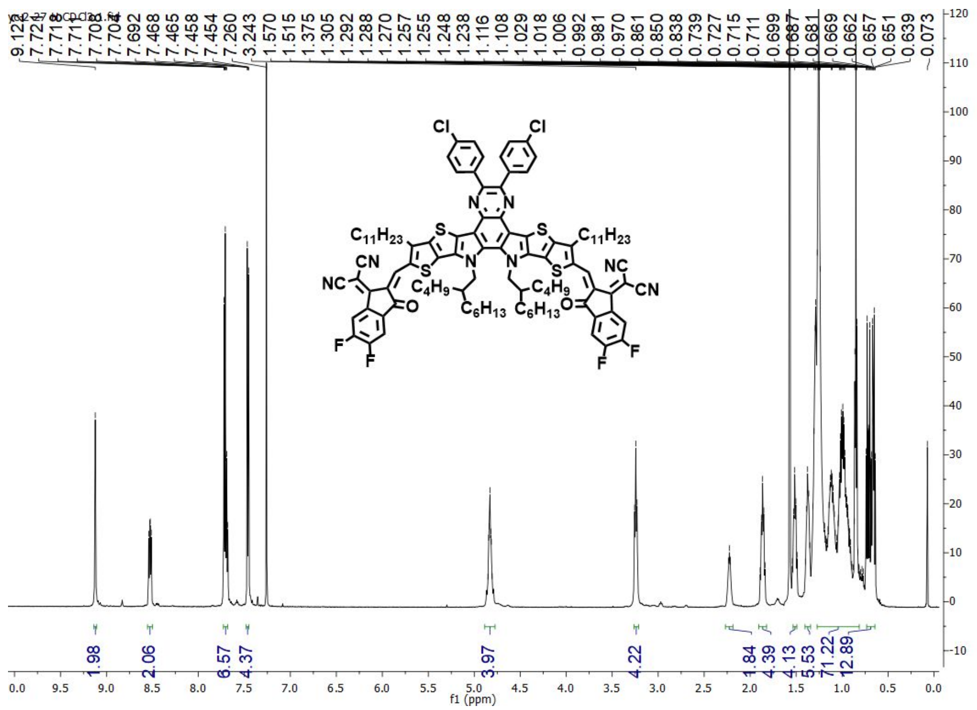

Figure S24 ^{1}H NMR spectrum of **Qx-PhCl** in $CDCl_3$.

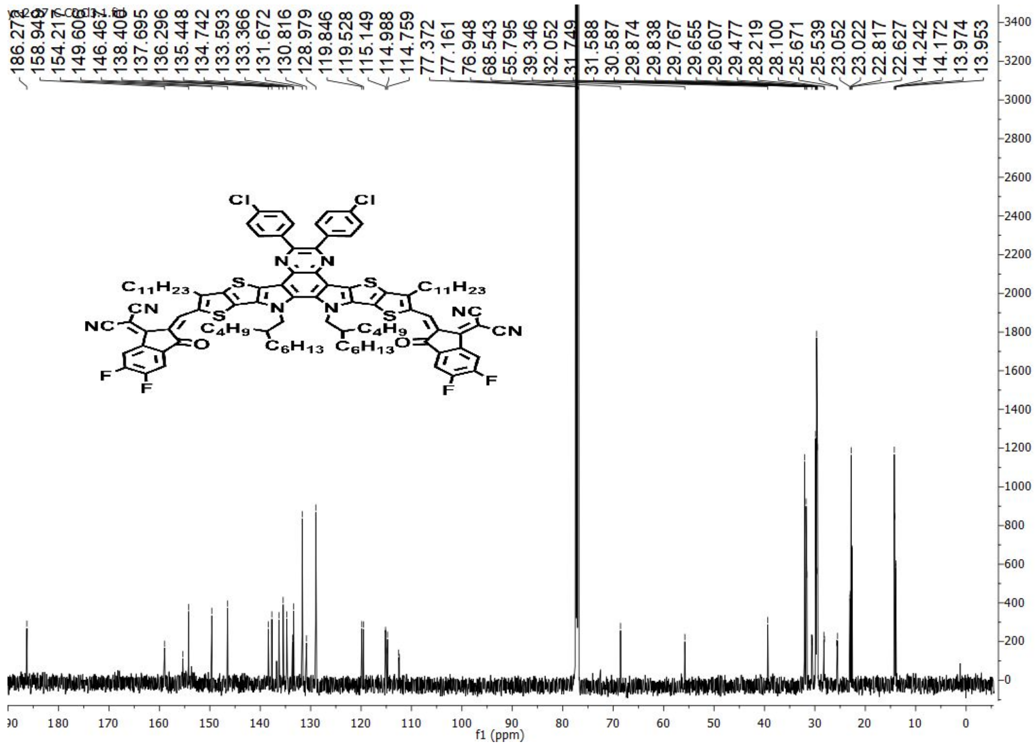

Figure S25 ^{13}C NMR spectrum of **Qx-PhCl** in $CDCl_3$.

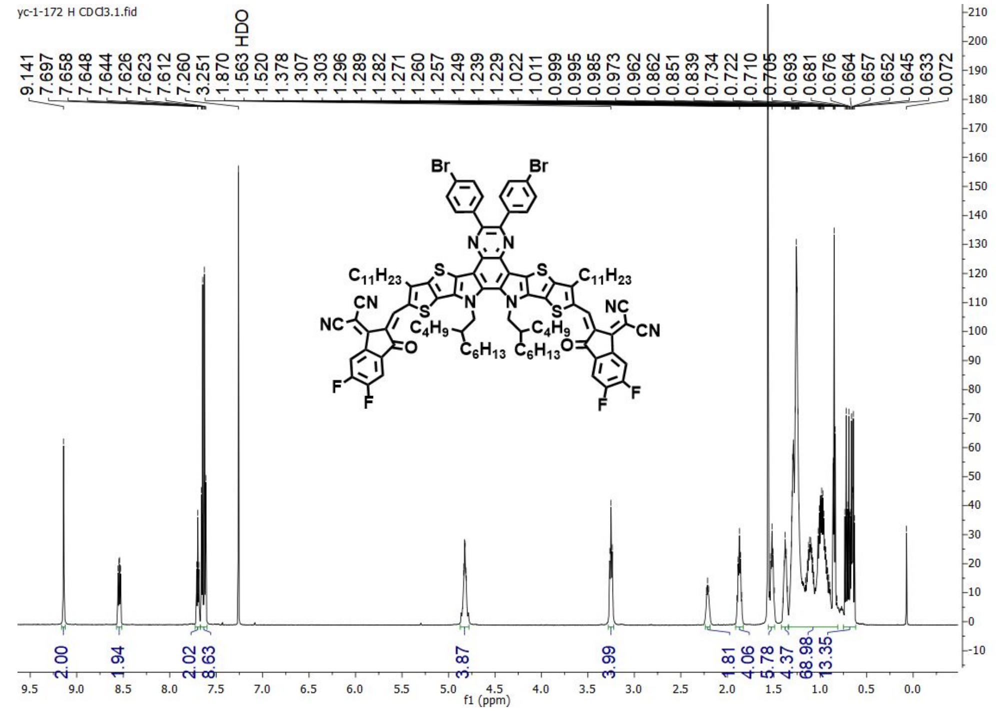

Figure S26 ^{1}H NMR spectrum of **Qx-PhBr** in $CDCl_3$.

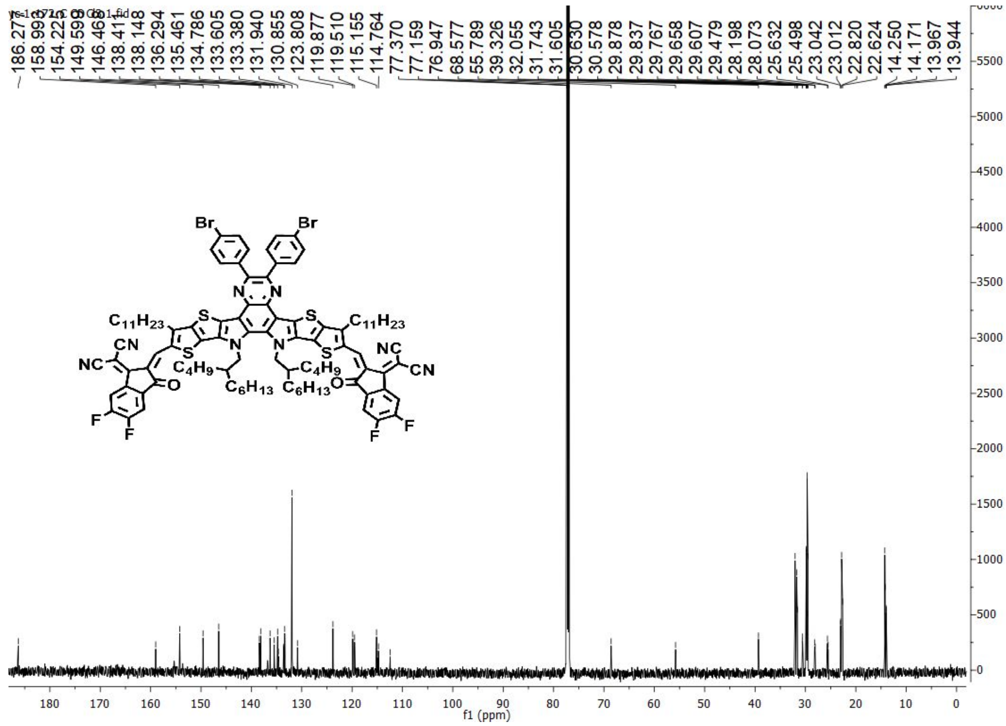

Figure S27 ^{13}C NMR spectrum of **Qx-PhBr** in $CDCl_3$.

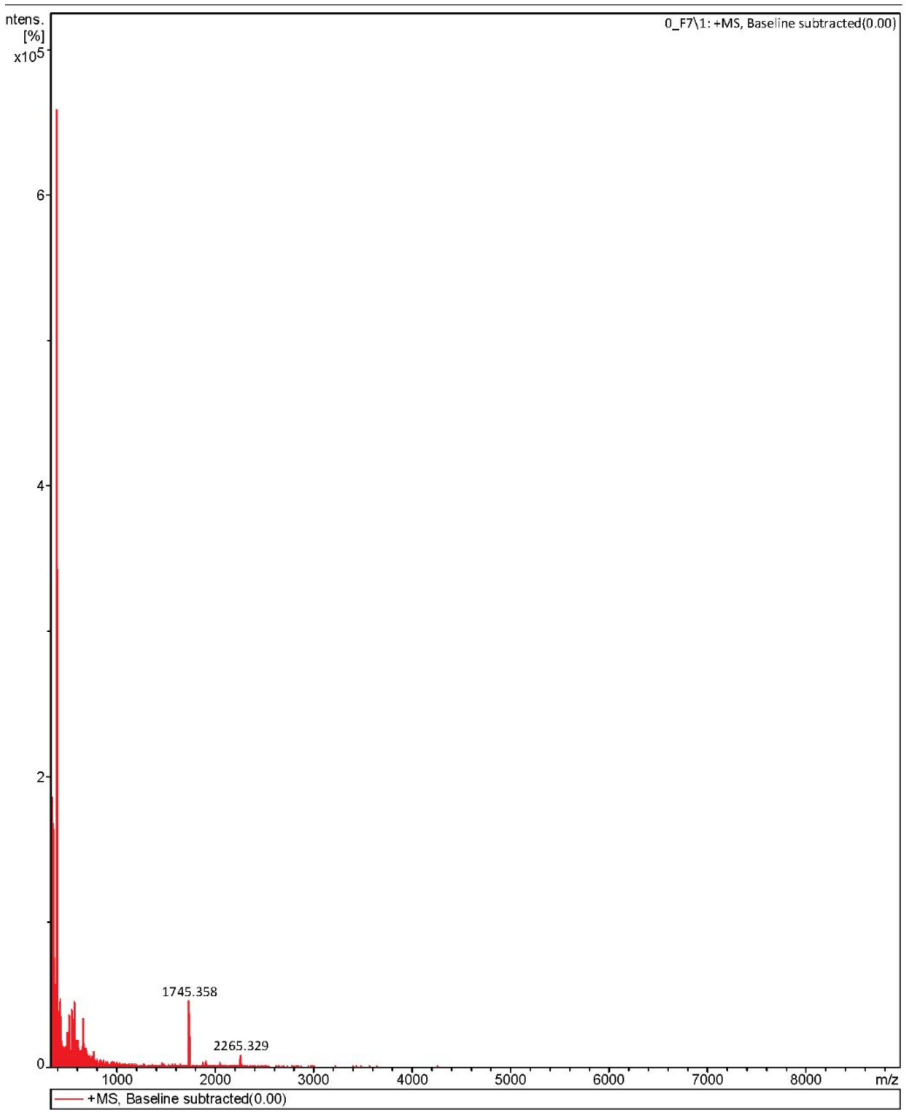

Figure S28 Mass spectrum of **Qx-PhF**.

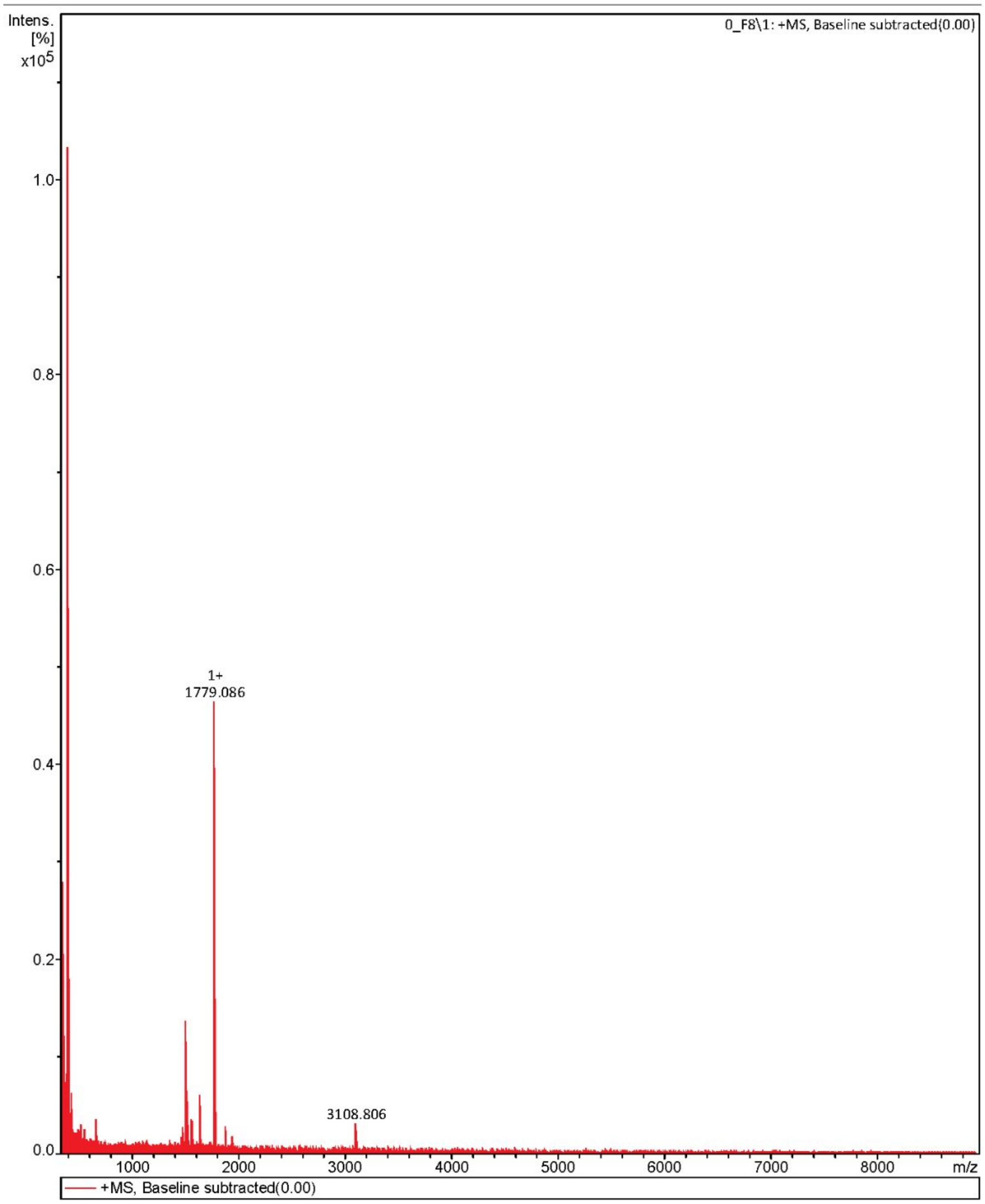

Figure S29 Mass spectrum of **Qx-PhCl**.

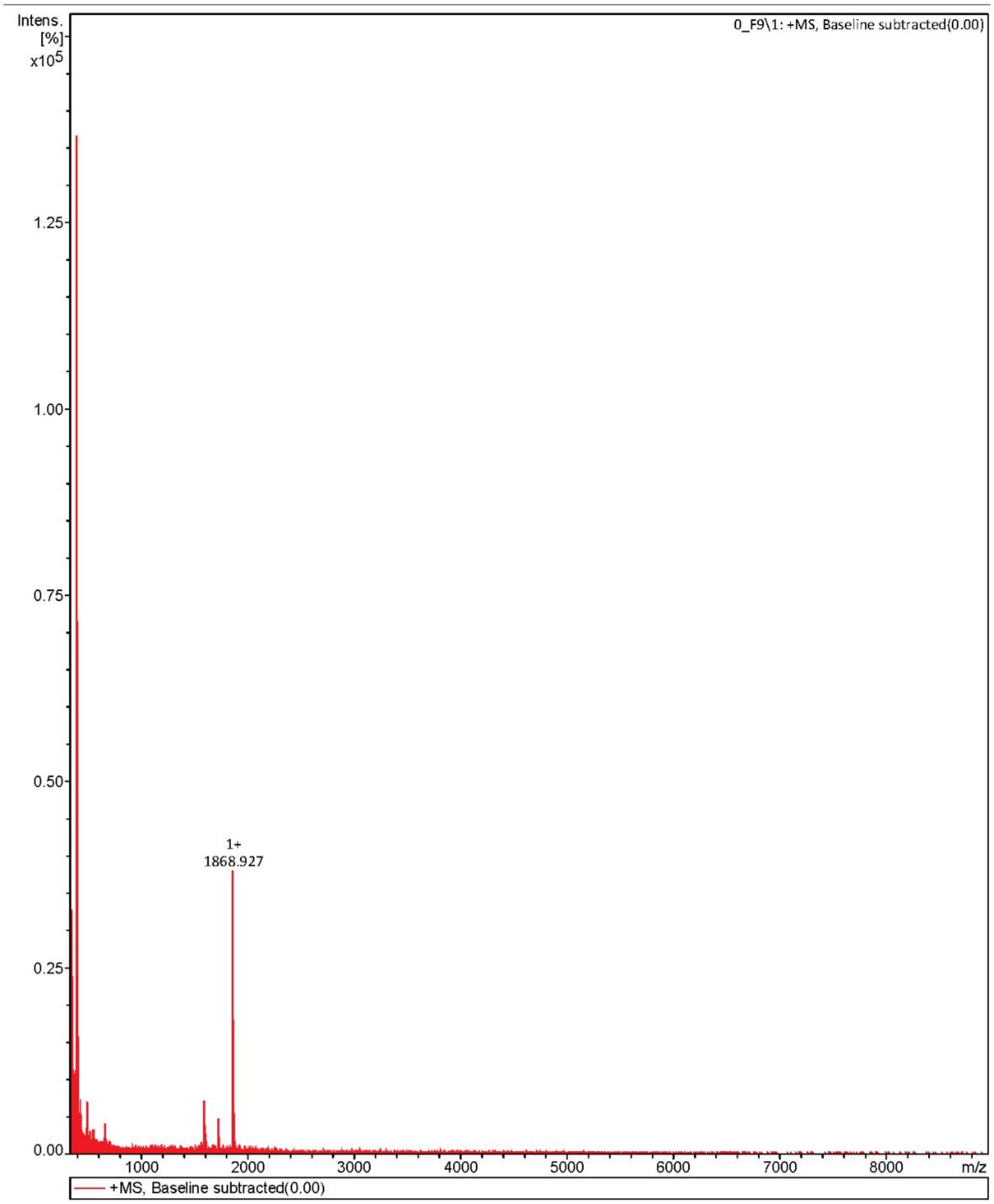

Figure S30 Mass spectrum of **Qx-PhBr**.

16. References

[1] J. Yuan, Y. Zhang, L. Zhou, G. Zhang, H.-L. Yip, T.-K. Lau, X. Lu, C. Zhu, H. Peng, P. A. Johnson, M. Leclerc, Y. Cao, J. Ulanski, Y. Li, Y. Zou, *Joule* **2019**, *3*, 1140-1151.

[2] Y. Shi, Y. Chang, K. Lu, Z. Chen, J. Zhang, Y. Yan, D. Qiu, Y. Liu, M. A. Adil, W. Ma, X. Hao, L. Zhu, Z. Wei, *Nat. Commun.* **2022**, *13*, 3256.

[3] M. J. Abraham, T. Murtola, R. Schulz, S. Páll, J. C. Smith, B. Hess, E. Lindahl, *SoftwareX* **2015**, *1*, 19-25.

[4] T. Lu, F. Chen, *Journal of Computational Chemistry* **2012**, *33*, 580-592.

[5] L. Martínez, R. Andrade, E. G. Birgin, J. M. Martínez, *J. Computational Chem.* **2009**, *30*, 2157-2164.

[6] L. Liu, F. Yu, D. Hu, X. Jiang, P. Huang, Y. Li, G. Tian, H. Lei, S. Wu, K. Tu, C. Chen, T. Gu, Y. Chen, T. Duan, Z. Xiao, *Energy Environ. Sci.* **2025**, *18*, 1722-1731.

[7] K. Tu, X. Jiang, G. Tian, L. Liu, Y. Chen, P. Huang, C. Chen, H. l. Lei, T. Duan, Z. Xiao, *J. Mater. Chem. C* **2025**, *13*, 7458-7461.
